# Real-world data emulating randomized controlled trials of non-vitamin K antagonist oral anticoagulants in patients with venous thromboembolism

**DOI:** 10.1186/s12916-023-03069-1

**Published:** 2023-09-29

**Authors:** Dongwon Yoon, Han Eol Jeong, Sohee Park, Seng Chan You, Soo-Mee Bang, Ju-Young Shin

**Affiliations:** 1 School of Pharmacy, Sungkyunkwan University, Suwon, South Korea; 2 Department of Biohealth Regulatory Science, Sungkyunkwan University, Suwon, South Korea; 3 Research Department of Practice and Policy, School of Pharmacy, University College London, London, UK; 4 Department of Biomedical Systems Informatics, Yonsei University College of Medicine, Seoul, Republic of Korea; 5 Division of Hemato-Oncology, Department of Internal Medicine, Seoul National University College of Medicine, Seoul National University Bundang Hospital, Seongnam, South Korea; 6 Samsung Advanced Institute for Health Sciences & Technology, Sungkyunkwan University, Seoul, South Korea

**Keywords:** Epidemiologic methods, Clinical trials, Anticoagulants, Factor Xa inhibitors, Venous thromboembolism

## Abstract

**Background:**

Emulating randomized controlled trials (RCTs) by real-world evidence (RWE) studies would benefit future clinical and regulatory decision-making by balancing the limitations of RCT. We aimed to evaluate whether the findings from RWE studies can support regulatory decisions derived from RCTs of non-vitamin K antagonist oral anticoagulants (NOACs) in patients with venous thromboembolism (VTE).

**Methods:**

Five landmark trials (AMPLIFY, RE-COVER II, Hokusai-VTE, EINSTEIN-DVT, and EINSTEIN-PE) of NOACs were emulated using the South Korean nationwide claims database (January 2012 to August 2020). We applied an active comparator and new-user design to include patients who initiated oral anticoagulants within 28 days from their VTE diagnoses. The prespecified eligibility criteria, exposure (each NOAC, such as apixaban, rivaroxaban, dabigatran, and edoxaban), comparator (conventional therapy, defined as subcutaneous heparin followed by warfarin), and the definition of outcomes from RCTs were emulated as closely as possible in each separate emulation cohort. The primary outcome was identical to each trial, which was defined as recurrent VTE or VTE-related death. The safety outcome was major bleeding. Propensity score matching was conducted to balance 69 covariates between the exposure groups. Effect estimates for outcomes were estimated using the Mantel–Haenszel method and Cox proportional hazards model and subsequently compared with the corresponding RCT estimates.

**Results:**

Compared to trial populations, real-world study populations were older (range: 63–69 years [RWE] vs. 54–59 years [RCT]), with more females (55–60.5% vs. 39–48.3%) and had a higher prevalence of active cancer (4.2–15.4% vs. 2.5–9.5%). The emulated estimates for effectiveness outcomes showed superior effectiveness of NOAC (AMPLIFY: relative risk 0.81, 95% confidence interval 0.70–0.94; RE-COVER II: hazard ratio [HR] 0.60, 0.37–0.96; Hokusai-VTE: 0.49, 0.31–0.78; EINSTEIN-DVT: 0.54, 0.33–0.89; EINSTEIN-PE: 0.50, 0.34–0.74), when contrasted with trials that showed non-inferiority. For safety outcomes, all emulations except for AMPLIFY and EINSTEIN-DVT yielded results consistent with their corresponding RCTs.

**Conclusions:**

This study revealed the feasibility of complementing RCTs with RWE studies by using claims data in patients with VTE. Future studies to consider the different demographic characteristics between RCT and RWE populations are needed.

**Supplementary Information:**

The online version contains supplementary material available at 10.1186/s12916-023-03069-1.

## Background

Randomized controlled trials (RCT) remain the gold standard for assessing the efficacy and safety of medical products for clinical and regulatory approval. However, the strict conditions required to demonstrate efficacy often limit the applicability of RCT results to routine clinical practice, referred to hereafter as the real world. To address this challenge, there is increasing interest in the use of non-interventional real-world data [1, 2]. However, concerns remain regarding the reliability and validity of estimates derived from real-world evidence (RWE) studies [3, 4]. To assess whether RWE studies using similar methodologies can provide supportive evidence for RCTs, it is necessary to calibrate RWE studies against the treatment effect of RCTs [5].

The introduction of non-vitamin K antagonist oral anticoagulants (NOACs), which have demonstrated non-inferior efficacy and safety compared to conventional therapy in patients with venous thromboembolism (VTE) [6–10], has dramatically shifted the therapeutic paradigm for VTE treatment. Accordingly, NOACs have become the preferred option over warfarin in acute and extended treatment phases [11]. Nevertheless, since patients with severe underlying diseases are generally excluded from trials, there is limited evidence on the use of NOACs in these patients. Furthermore, potential conflicts of interest among trial sponsors may make it difficult to evaluate the comparative efficacy of different NOACs. In light of these shortcomings of RCT, RWE studies could serve as an alternative to assess both the treatment effects of NOACs and their comparative effectiveness in real-world practice.

Given the possibility of utilizing RWE for regulatory decision-making, calibrating the estimates derived from real-world data sources against RCT findings would provide supportive evidence to better understand the validity of RWE studies. Therefore, we compared RWE studies that emulated effectiveness outcomes with the efficacy results of corresponding RCTs in the NOAC-VTE setting.

## Methods

### Data sources

We used the Health Insurance Review and Assessment (HIRA) database of South Korea from January 1, 2012, to August 31, 2020. This database contains all Korean healthcare utilization information, including diagnoses, prescriptions, and surgical procedures. Patient data remains anonymous using de-identified keys [12]. The HIRA database covers a population of > 50 million, and all citizens are continuously enrolled unless they are ineligible due to emigration or death. Hence, comprehensive information on personal characteristics and healthcare utilization based on reimbursed claims of inpatient, outpatient, and emergency department visits is available for assessment. HIRA contains data on diagnoses, procedures, length of hospitalization, and prescribed medications including length of prescription, dose, route of administration, and costs. All procedures and prescriptions (mapped to the Anatomical Therapeutic Chemical classification system) are coded using domestic codes. Diagnoses are coded using the Korean Standard Classification of Diseases, 7th revision, and a modified version of the International Classification of Diseases, 10th revision (ICD-10). In a previous validation study, the positive predictive value (PPV) of diagnosis codes in claims data was reported to be 82%. This was calculated by comparing the diagnoses obtained from electronic medical records, which served as the gold standard for validation.

### Study design and cohort

We conducted an active comparator, new-user, propensity score-matched, nationwide cohort study to emulate five pivotal NOAC (apixaban, dabigatran, edoxaban, rivaroxaban) trials in patients with VTE. The studies emulated included AMPLIFY (Apixaban for the Initial Management of Pulmonary Embolism and Deep-Vein Thrombosis as First-Line Therapy) [6], RE-COVER II (Phase III Study Testing Efficacy & Safety of Oral Dabigatran Etexilate vs. Warfarin for 6-month Treatment for Acute Symptomatic Venous Thromboembolism) [7], EINSTEIN-DVT (Oral Direct Factor Xa Inhibitor Rivaroxaban in Patients With Acute Symptomatic Deep Vein Thrombosis) [9], EINSTEIN-PE (Oral Direct Factor Xa Inhibitor Rivaroxaban in Patients With Acute Symptomatic Pulmonary Embolism) [10], and Hokusai-VTE (Comparative Investigation of Low Molecular Weight Heparin/Edoxaban Tosylate Versus Heparin/Warfarin in the Treatment of Symptomatic Deep-Vein Blood Clots and/or Lung Blood Clots) [8]. The RCTs included in our study were selected because they involve an active comparator setting, directly comparing each NOAC with conventional therapy (subcutaneous heparin followed by warfarin). This active comparator setting is beneficial as it improves the comparability between the two treatment groups, thereby enhancing the validity of real-world evidence (RWE) by increasing the likelihood of emulation.

For each trial, an emulation cohort was constructed separately, and the study period was defined as the date of reimbursement of each NOAC indicated for recurrent VTE (rivaroxaban, January 2013; apixaban/dabigatran, May 2015; edoxaban, February 2016) to the last available date in the database (August 31, 2020). In the process of creating the emulation cohort (RWE cohort) using the HIRA database, we implemented the same prespecified inclusion and exclusion criteria that were utilized in the corresponding RCT as closely as possible to ensure comparability between the RCTs and the RWE study cohorts (details of emulating each trial, including protocol and eligibility criteria, are available at the “Availability of data and materials” section of the manuscript). Each cohort comprised adult patients newly initiating treatment with individual NOAC or warfarin (index date) with a prior diagnosis of DVT or PE in the primary or secondary position within 30 days before and including the index date from an inpatient or emergency department (ED) setting. New use was defined as no filled prescription of oral anticoagulant in the 180-day period preceding the index date. All exclusion criteria were applied in the 180 days before and including the index date, unless specified otherwise in the protocol of each trial (detailed study designs are available in Additional file 1: Fig. S1-S5).

### Outcomes and follow-up

In the EINSTEIN-DVT and PE emulations, the primary outcome was recurrent VTE, defined as a composite of DVT or PE using ICD-10 diagnostic codes in the primary position of inpatient claims accompanying imaging procedures (ultrasonography, computer tomography scan, venography) and a previously validated algorithm that showed a PPV of 83% was used [13, 14]. In AMPLIFY, RE-COVER II, and Hokusai-VTE emulations, the primary outcome was a composite of recurrent VTE and VTE-related death. The detailed diagnosis and procedure codes are shown in Additional file 1: Table S1.

Patients were followed from the index date until the earliest outcome occurrence: switch to another oral anticoagulant (e.g., apixaban to edoxaban or warfarin), treatment discontinuation (> 10-day gap between last filled prescription and start of subsequent prescription), in-hospital death, or the end of the study period. Considering the likelihood of lower treatment adherence in real-world versus trial settings, the “as-treated” approach (analog of per-protocol design of trials) was selected as the primary analysis to estimate relative hazards while patients were receiving treatment. In the AMPLIFY emulation, treatment of an episode of VTE with thrombectomy, insertion of a caval filter, or use of fibrinolytic agents (streptokinase, alteplase, tenecteplase) were regarded as additional censoring criteria in order to reflect the original trial protocol.

### Potential confounders

We assessed 69 potential confounders to adjust for underlying differences and to obtain comparability between the groups in the RWE study as a proxy for mimicking baseline randomization. Age and sex were assessed at the index date, and unless specified otherwise, other covariates were assessed from 180 days before and including the index date. Covariates included the following: active cancer, defined as a diagnosis of cancer (other than non-melanoma skin cancer) or ongoing treatment for cancer (chemotherapy, radiation therapy, and surgical procedure) [15]; history of disease diagnoses (previous VTE, stroke, chronic kidney disease); healthcare-related procedures or tests (cardiovascular stress test, echocardiogram, cardiac biomarker tests, international normalized ratio, bleeding time tests); concomitant medications (meglitinides, alpha-glucosidase inhibitors, thiazide, beta-blockers); indicators of health status (HAS-BLED score, CHA_2_DS_2_-VASc score, Charlson comorbidity index); and other relevant variables. Details on potential confounders are listed in Additional file 1: Table S2.

### Statistical analysis

Baseline characteristics of exposed and comparator groups were summarized as frequencies with proportions for categorical variables and as means with standard deviations for continuous variables. The propensity score (PS) was estimated in each emulated cohort to minimize the systematic differences in the baseline characteristics between the two groups. All 69 aforementioned covariates were included in the logistic regression model to estimate the probability of receiving treatment, conditional on their covariates. As each NOAC was approved at various times in Korea, substantial biases are likely to arise from the prescribing trend or channeling over time [16, 17]. To minimize this bias, we implemented a 1:1 propensity score nearest-neighbor matching with a caliper of 0.01 on the propensity score scale, stratified into two calendar year intervals [5, 18]. Differences in baseline covariates between the two groups were evaluated before and after the propensity score matching using an absolute standardized difference with a value of > 0.1 indicating a significant difference (results of PS distribution are available in Additional file 1: Fig. S6-S10).

Within the matched cohort, we estimated the median follow-up and incidence of outcomes per 100 person-years as well as the corresponding hazard ratios (HR) with 95% confidence intervals (CI) using a Cox proportional hazard model. Given that the AMPLIFY trial used the Mantel–Haenszel method to estimate relative risk (RR), stratified by the type of VTE (DVT or PE), we also used this method for the AMPLIFY emulation only. For each emulation, we summarized and compared selected baseline characteristics (age, sex, type of VTE, previous VTE, and active cancer) between the RWE emulations and corresponding RCTs. There were no missing variables in any of the emulation analyses. All analyses were conducted using the SAS Enterprise Guide (version 7.1; SAS Institute Inc., Cary, USA). A two-tailed *p*-value < 0.05 was considered statistically significant.

### Exploratory analysis for safety assessment

We repeated our emulations for safety outcomes and compared them to established safety profiles of current knowledge within the trial-mimicking population. For the exploratory outcome, major bleeding was considered as a safety outcome and defined as a composite of intracranial, gastrointestinal, and other bleeding verified using ICD-10 codes in the primary position of inpatient claims. The diagnostic codes in the primary position during hospitalization showed a PPV of 92% for gastrointestinal bleeding [19]. The study also reported a PPV of 81.4% for intracranial bleeding with an imaging diagnosis. In terms of intracranial bleeding, imaging procedures combined with diagnostic codes were regarded as true intracranial bleeding to increase the PPV of the diagnosis. Detailed ICD-10 diagnosis and procedure codes are shown in Additional file 1: Table S3.

### Sensitivity analysis

A sensitivity analysis was conducted for all outcomes to examine the robustness of the main findings. First, the “intention-to-treat (ITT)” approach was implemented, which investigated the efficacy of the randomized assigned treatment, regardless of treatment adherence [20, 21]. To mimic this approach, patients were followed up from the index date until outcome occurrence, in-hospital death, the end of the study period, or a pre-specified time interval from each RCT (AMPLIFY, RE-COVER II: 180 days; EINSTEIN-DVT/PE, Hokusai-VTE: 365 days). Second, we conducted asymmetrical trimming in the pre-matched cohort to exclude patients with propensity score values below 2.5% in the treatment group and above 97.5% of the comparator group [22, 23] and estimated HRs with 95% CIs using Cox proportional hazards models adjusted for propensity score deciles.

### Agreement metrics between RCT-RWE findings

To compare the findings from RCTs and RWE studies, we adopted the same agreement metrics developed and used by the Randomized Controlled Trials Duplicated Using Prospective Longitudinal Insurance Claims: Applying Techniques of Epidemiology (RCT DUPLICATE) initiative [5]. The first metric was regulatory agreement (RA), which quantifies the extent to which the RWE study would suggest the same regulatory decision as the trial and was defined as whether the estimated effect in the RWE study did not exceed the prespecified non-inferiority margin of the corresponding RCT [24]. If the result of the non-inferiority trial did not exceed the prespecified non-inferiority margin, the effect estimate of the corresponding RWE study would need to yield the same non-inferiority (including superiority) to meet RA [25]. For example, if the RCT estimate was 0.89 (95% CI 0.76–1.21; margin 1.8) and the RWE estimate was 0.78 (95% CI 0.63–0.89), the RA criterion was considered met. The second metric was the estimate agreement (EA), defined as whether the RWE estimate lay within the 95% CI of the RCT estimate [24, 25]. The last metric, standardized difference (SD), estimates the extent and direction of difference in findings between the RCT and the corresponding RWE study.

## Results

### Baseline characteristics between RCTs and RWE studies

In this active comparator, new-user, propensity score-matched analysis, the emulation cohort for our study included the following number of study participants in each treatment arm (for each NOAC and warfarin) after applying the eligibility criteria: 1753 for AMPLIFY emulation, 1226 for RE-COVER II emulation, 1505 for Hokusai-VTE emulation, 2135 for EINSTEIN-DVT emulation, and 2801 for EINSTEIN-PE emulation. Across the five trials, the mean age range was 54–58 years, whereas 63–69 years in RWE studies (Table 1; Additional file 1: Table S4-S14). Despite applying the same inclusion and exclusion criteria, the mean age of the study population in each emulation was substantially higher than that of the corresponding trial. Likewise, the proportion of each sex was also considerably different between the RCTs and RWE studies, with males being more prevalent in trials (range 51.7–61.0%) than in RWE studies (range 39.5–45.0%). Except for the EINSTEIN-DVT and PE trials that enrolled patients by subtype of VTE, the types of VTE revealed contrasting features across all emulations. While DVT was predominant in the AMPLIFY, RE-COVER II, and Hokusai-VTE trials, PE was more prevalent in each corresponding emulation. Previous VTE was similar between RCTs and RWE studies, although the EINSTEIN-DVT and PE trials showed modest differences, especially in the EINSTEIN-DVT trial (19.4 [RCT] vs. 24.7% [RWE] for rivaroxaban users; 19.2 [RCT] vs. 24.2% [RWE] for warfarin users). While the RE-COVER II and Hokusai-VTE trials showed comparable proportions in the presence of active cancer between RCT and RWE studies, a considerably higher proportion of active cancer was found in RWE studies than in RCTs of AMPLIFY and EINSTEIN studies. Given that the RE-COVER II trial used a longer assessment period (5 years) to define active cancer, the difference between RWE and RCT in the proportion of active cancer would likely be higher in RE-COVER II.

**Table 1:** Baseline characteristics of RCTs and the corresponding emulation of RWE studies

Variables	AMPLIFY		RE-COVER II		Hokusai-VTE		EINSTEIN-DVT		EINSTEIN-PE	
	**Apixaban**	**Warfarin**	**Dabigatran**	**Warfarin**	**Edoxaban**	**Warfarin**	**Rivaroxaban**	**Warfarin**	**Rivaroxaban**	**Warfarin**
**Age, mean ± SD, years**										
RCT	57.2 ± 16.0	56.7 ± 16.0	54.7 ± 16.2	55.1 ± 16.3	55.7 ± 16.3	55.9 ± 16.2	55.8 ± 16.4	56.4 ± 16.3	57.9 ± 7.3	57.5 ± 7.2
RWE	68.9 ± 17.0	68.9 ± 16.4	68.0 ± 16.3	68.0 ± 16.3	68.8 ± 16.1	68.8 ± 16.1	63.3 ± 16.7	62.9 ± 17.1	69.0 ± 15.5	69.0 ± 15.2
**Male, %**										
RCT	58.3	59.1	61.0	60.2	57.3	57.2	57.4	56.3	54.1	51.7
RWE	42.4	40.8	41.0	40.9	39.9	39.5	44.1	45.0	40.5	41.7
**DVT only, %**										
RCT	65.0	65.9	68.5	67.8	59.9	59.5	98.7	98.8	0	0
RWE	33.9	34.0	29.5	30.6	34.3	34.4	100	100	0	0
**PE with or without DVT, %**										
RCT	34.6	33.5	31.4	32.2	40.1	40.5	0.69	0.64	100	100
RWE	66.1	66.0	70.5	69.4	65.7	65.6	0	0	100	100
**Previous VTE, %**										
RCT	17.2	15.1	19.3	15.8	19.0	17.9	19.4	19.2	18.8	20.3
RWE	18.0	17.9	18.8	18.2	19.6	18.3	24.7	24.2	14.8	15.4
**Active cancer, %**										
RCT	2.5	2.8	3.9	3.9	9.2	9.5	6.8	5.2	4.7	4.5
RWE	12.2	11.0	4.2	4.4	10.6	10.4	11.6	11.0	15.0	15.4
**Asian fraction, %**^a^										
RCT	8.4	8.4	20.9^a^	20.9^a^	21.0	20.9	12.2	12.3	6.0	5.9

*Abbreviations*: *RCT* randomized controlled trial, *RWE* real-world evidence, *SD* standard deviation, *DVT* deep vein thrombosis, *PE* pulmonary embolism, *VTE* venous thromboembolism

^a^Although information on race/ethnicity is unavailable in the HIRA database, the Asian fraction would be estimated to be approximately 99.9% (only 60,000–70,000 patients are non-Asian among the whole population in the Korean national health insurance system), the values of RE-COVER II were not reported separately in each intervention group

### Comparison of effect estimates between RCTs and RWE studies

**Fig. 1:**
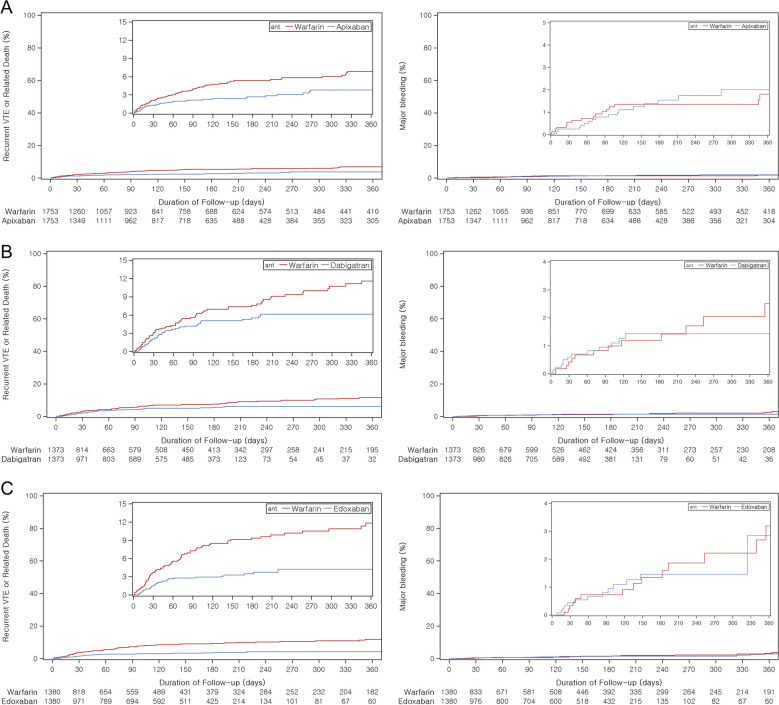

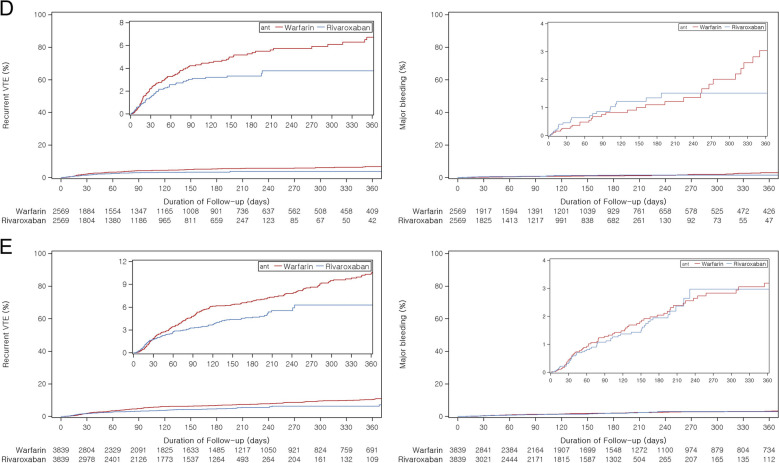
Cumulative incidence of the effectiveness and safety outcomes in emulation studies. **A** AMPLIFY emulation **B** RE-COVER II emulation **C** Hokusai-VTE emulation **D** EINSTEIN-DVT emulation **E** EINSTEIN-PE emulation

Event rates for effectiveness and safety outcomes between trials and their emulations could not be directly compared because these data were not reported from all trials, although the proportion of effectiveness outcomes was comparable (Table 2). While all RCT estimates for effectiveness outcomes showed non-inferiority, their corresponding RWE estimates all showed superior effectiveness: AMPLIFY (RR 0.81, 95% CI 0.70–0.94), RE-COVER II (HR 0.60, 95% CI, 0.37–0.96), Hokusai-VTE (HR 0.49, 95% CI, 0.31–0.78), EINSTEIN-DVT (HR 0.54, 95% CI, 0.33–0.89), and EINSTEIN-PE (HR 0.50, 95% CI, 0.34–0.74). Kaplan–Meier plots showing trends in event occurrence demonstrate superior effectiveness for the primary outcome (Fig. 1).

**Table 2:** Comparison of study sizes, proportion of events, and effect estimates between RCTs and RWE studies

Trial emulation	RCT		RWE			RCT	RWE
	**Patients**	**Events (%)**	**Patients**	**Events (%)**	**Incidence rate**^**a**^	**Effect estimates**^**b**^** (95% CI)**	
**AMPLIFY**							
Recurrent VTE or related death							
Warfarin	2635	71 (2.69)	1,753	78 (4.45)	6.33	1.00 (ref)	1.00 (ref)
Apixaban	2609	59 (2.26)	1,753	50 (2.85)	4.63	0.84 (0.60–1.18)	0.81 (0.70 to 0.94)
Major bleeding							
Warfarin	2689	49 (1.82)	1753	20 (1.14)	1.60	1.00 (ref)	1.00 (ref)
Apixaban	2676	15 (0.56)	1753	20 (1.14)	1.85	0.31 (0.17–0.55)	0.99 (0.73 to 1.37)
**RE-COVER II**							
Recurrent VTE or related death							
Warfarin	1289	28 (2.17)	1226	44 (3.59)	7.85	1.00 (ref)	1.00 (ref)
Dabigatran	1279	30 (2.34)	1226	29 (2.37)	7.58	1.08 (0.64–1.80)	0.60 (0.37 to 0.96)
Major bleeding							
Warfarin	1289	22 (1.70)	1226	9 (0.73)	1.57	1.00 (ref)	1.00 (ref)
Dabigatran	1279	15 (1.17)	1226	6 (0.49)	1.53	0.69 (0.36–1.32)	0.62 (0.22 to 1.77)
**Hokusai-VTE**							
Recurrent VTE or related death							
Warfarin	4122	146 (3.54)	1505	48 (3.19)	8.31	1.00 (ref)	1.00 (ref)
Edoxaban	4118	130 (3.15)	1505	28 (1.86)	5.51	0.89 (0.70–1.13)	0.49 (0.31 to 0.78)
Major bleeding							
Warfarin	4122	66 (1.60)	1505	13 (0.86)	2.23	1.00 (ref)	1.00 (ref)
Edoxaban	4118	56 (1.35)	1505	8 (0.53)	1.56	0.84 (0.59–1.21)	0.61 (0.25 to 1.51)
**EINSTEIN-DVT**							
Recurrent VTE							
Warfarin	1718	51 (2.96)	2135	50 (2.34)	4.22	1.00 (ref)	1.00 (ref)
Rivaroxaban	1731	36 (2.07)	2135	23 (1.08)	3.60	0.69 (0.44–1.04)	0.54 (0.33 to 0.89)
Major bleeding							
Warfarin	1711	20 (1.16)	2135	17 (0.80)	1.40	1.00 (ref)	1.00 (ref)
Rivaroxaban	1718	14 (0.81)	2135	12 (0.56)	1.87	0.65 (0.33–1.30)	1.62 (0.70 to 3.72)
**EINSTEIN-PE**							
Recurrent VTE							
Warfarin	2413	44 (1.82)	2801	91 (3.25)	5.42	1.00 (ref)	1.00 (ref)
Rivaroxaban	2419	50 (2.06)	2801	38 (1.36)	3.83	1.12 (0.75–1.68)	0.50 (0.34 to 0.74)
Major bleeding							
Warfarin	2405	52 (2.16)	2801	37 (1.32)	2.16	1.00 (ref)	1.00 (ref)
Rivaroxaban	2412	26 (1.07)	2801	16 (0.57)	1.60	0.49 (0.31–0.79)	0.65 (0.35 to 1.21)

*Abbreviations*: *CI* confidence interval, *VTE* venous thromboembolism

^a^Crude incidence rate per 100 person-years. The incidence rates were not reported in the RCTs

^b^Mantel–Haenszel relative risk was estimated following the protocol of the AMPLIFY trial; hazard ratio was estimated, unless specified otherwise

### Exploratory outcome and sensitivity analysis

All RWE emulations except for EINSTEIN-DVT yielded consistent results for estimates direction with the safety outcome of RCTs, by demonstrating NOAC-favorable results over conventional therapy: AMPLIFY (RR 0.99, 95% CI 0.73–1.37), RE-COVER II (HR 0.62, 95% CI 0.22–1.77), Hokusai-VTE (HR 0.61, 95% CI 0.25–1.51), and EINSTEIN-PE (HR 0.65, 95% CI 0.35–1.21). While AMPLIFY (RR 0.31, 95% CI 0.17–0.55) and EINSTEIN-PE (HR 0.49, 95% CI 0.31–0.79) trials found a significantly lower risk of major bleeding, the results of the corresponding emulations showed the same direction but did not reach statistical significance. Meanwhile, the results of the RE-COVER II and Hokusai-VTE trials were comparable across the RCT and RWE studies. Effect estimate findings from EINSTEIN-DVT trial and corresponding emulation were substantially different: EINSTEIN-DVT trial (HR 0.65, 95% CI 0.33–1.30) vs. the corresponding emulation (HR 1.62, 95% CI 0.70–3.72).

For sensitivity analysis, the results of the intention-to-treat approach were consistent for both the primary and exploratory outcomes (Additional file 1: Table S14). Overall, the estimates of the intention-to-treat approach yielded conservative results that were closer to null compared to the main analyses. In this setting, the results of EINSTEIN-DVT were consistent with the safety outcome of RCT (HR 0.84, 95% CI 0.49–1.42). In other sensitivity analyses, the alternative PS method showed similar results for both primary and exploratory outcomes (Additional file 1: Table S15). The results for both outcomes were largely comparable and yielded relatively narrow confidence intervals.

### Evaluation of agreements between RCTs and RWE studies

**Fig. 2:**
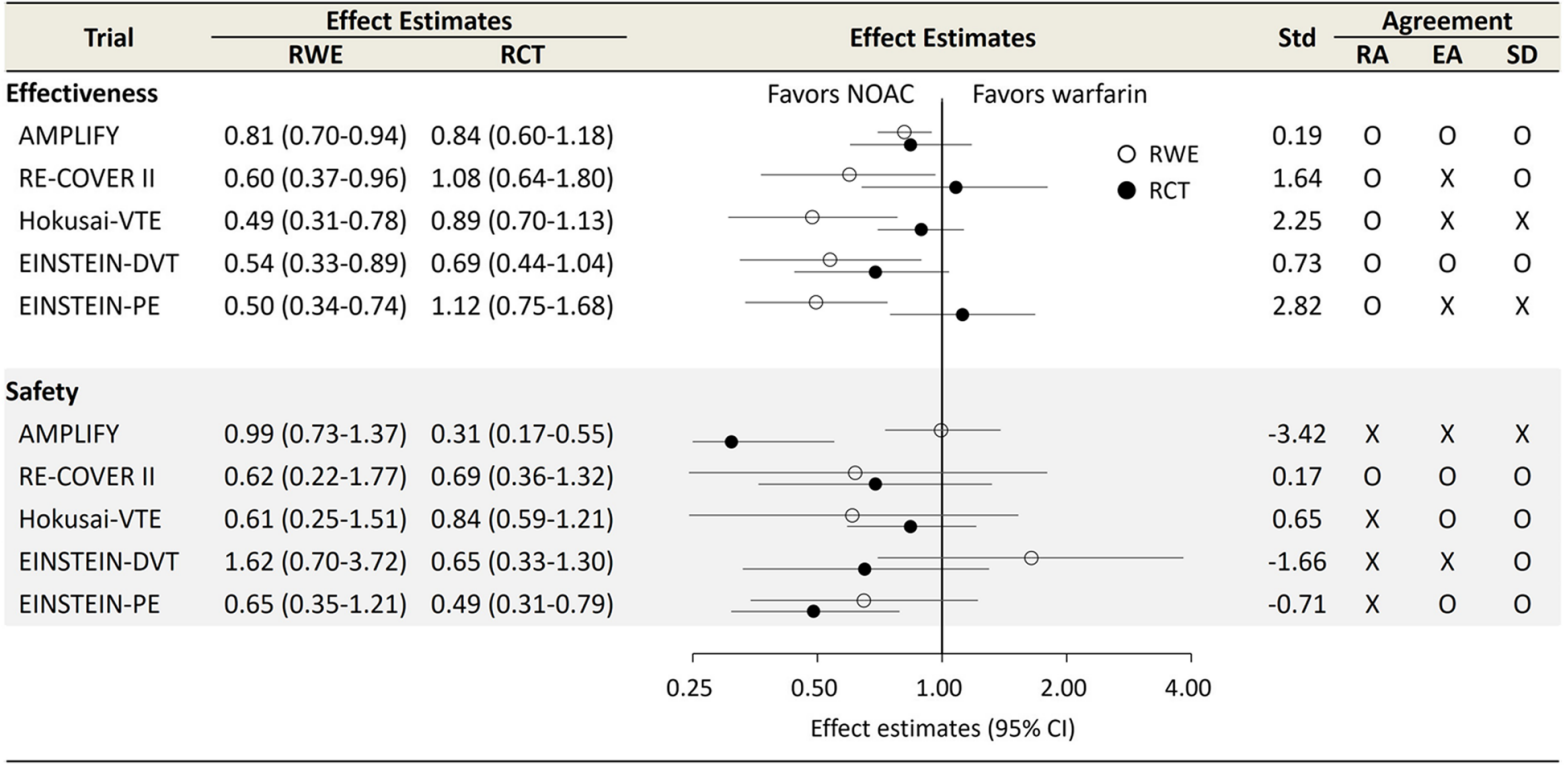
Effect estimates and evaluation of agreement between findings from RCTs and corresponding RWE studies. *Abbreviations*: RCT, randomized controlled trial; RWE, real-world evidence; Std, standardized difference; RA, regulatory agreement; EA, estimate agreement; SD, standardized differences; HR, hazard ratio; CI, confidence interval. *Note*: AMPLIFY yielded relative risk using the Mantel–Haenszel method; therefore, the corresponding emulation also yielded relative risk using the same method. White circles indicate HR from RWE studies, and black circles indicate HR from RCT. The predefined non-inferiority margin was 1.8 for AMPLIFY and RE-COVER II trial, 1.5 for Hokusai-VTE trial, and 2.0 for EINSTEIN-DVT and EINSTEIN-PE trial

For the effectiveness outcome, RA was achieved for all emulations (Fig. 2). In line with the results of all RCTs that found non-inferiority of effect estimates within the predefined margin, all emulations also found non-inferior effects of NOAC versus warfarin but also found superior effectiveness. The EA was met for two emulations (AMPLIFY and EINSTEIN-DVT). The estimates of Hokusai-VTE and EINSTEIN-PE showed a statistically significant difference between the RCT and its emulation (SD = 2.25 [Hokusai-VTE]; SD = 2.82 [EINSTEIN-PE]). For the safety outcome, the overall effect estimates were consistent with the current knowledge of the safety profiles of NOACs. The RA was met in RE-COVER II, while it was not met in Hokusai-VTE, as the upper limit of 95% CI exceeded the predefined non-inferiority margin from the Hokusai-VTE trial (non-inferiority margin: 1.5). The EA was met in RE-COVER II, Hokusai-VTE, and EINSTEIN-PE. AMPLIFY was the only trial that showed a significant difference in safety outcomes between RCTs and corresponding emulations (SD, − 3.42).

## Discussion

In this study, five pivotal trials of NOAC in patients with VTE were emulated using Korea’s nationwide claims data for comparison with RCTs. The baseline characteristics of each trial and corresponding emulations were generally different; real-world populations were older, more female, and had a higher prevalence of active cancer. We found that NOACs are not only non-inferior to warfarin therapy, as proven in all landmark trials [6–10], but are likely superior in terms of effectiveness. Based on the extent of agreement between the RCTs and RWE studies, RA was achieved for all five emulations, EA for two emulations, and SD for three emulations. Regarding individual trials, AMPLIFY and EINSTEIN-DVT met all the binary criteria (RA, EA, SD), whereas Hokusai-VTE and EINSTEIN-PE met only RA.

Overall, the extent of agreement between RCTs and the corresponding RWE studies was best for RA (met 5 out of 5) and SD (met 3 out of 5) as opposed to EA, likely because RWE estimates showed a significantly lower risk of the effectiveness outcome in each emulation than those of RCTs. In comparison with the results of emulation of RCTs using non-randomized databases conducted by the RCT DUPLICATE initiative [25], our study presented very similar findings in terms of RA (comparable to the statistical significance of the previous study) and SD. Particularly, for EINSTEIN-DVT and EINSTEIN-PE, our results aligned with the previous study regarding RA, EA, and SD. However, in our study, we found different results for EA because the effect estimates were generally more favorable for NOACs compared to the results of the RCT and the previous study.

Despite attempts to replicate the key features of each trial as closely as possible, such as inclusion/exclusion criteria, exposures, and outcomes, inherent limitations of the claims data made exact emulation impossible. Heterogeneous characteristics of the study population, exposure adherence and follow-up, and racial and ethnic differences between RCTs and the Korean RWE could be potential explanations for the observed differences [26]. First, different baseline population characteristics between RCT and RWE studies may cause the effect estimate to be a different direction or significance. In support, RWE studies had more females, an older population, and higher cancer prevalence than RCTs. Previous studies have explored the impact of sex on outcomes associated with oral anticoagulants, leading to diverse findings without a clear consensus in patients with VTE [27–30]. However, given the higher risk of bleeding in women observed in the prior meta-analysis [28] and the proportional differences in sex between our emulation cohort and the corresponding RCT population, the potential impact of sex differences in effect estimates could not be ruled out. Differential age, a prominent factor in VTE incidence [31], could also have impacted the effect estimate, as increasing age is associated with increased comorbidity and vascular elasticity. Moreover, the comparative efficacy and safety of NOACs stratified by age showed a shift in trial estimates, favoring NOACs [32, 33]. Another possible difference between RCTs and RWE studies could be owed to the distribution of active cancer, especially in AMPLIFY, EINSTEIN-DVT, and EINSTEIN-PE. One systematic review reported a trend in favor of NOACs for the efficacy outcome in patients with active cancer by showing preventive effects with NOACs compared to patients with non-active cancer [34]. Although subgroup findings for patients with active cancer were not available in the AMPLIFY trial, the EINSTEIN-DVT, EINSTEIN-PE, RE-COVER, and Hokusai-VTE trials consistently showed more preventive effects in patients with active cancer. In this context, the heterogeneous distribution of effect modifiers in each emulation could have affected the accurate calibration of RWE studies against RCTs in our NOAC-VTE setting [35].

The treatment quality of warfarin is an important factor in patients with VTE or atrial fibrillation. In East Asian populations, including Korean patients, the recommended warfarin maintenance target often sets a lower range of INR due to a higher risk of bleeding even with similar INR levels compared to Caucasian populations [36, 37]. While the target INR of 2.0–3.0 has well been established in Caucasian populations, Asian physicians conventionally tend to adhere to a target INR of 1.6–2.6 due to bleeding concerns [38], resulting in a relatively lower mean time in the therapeutic range (TTR) for warfarin compared to RCTs. This difference in treatment quality could potentially impact the effect estimates between RWE and RCTs. Our findings of the superior effectiveness of NOACs in emulation trials compared to RCTs may be supported by the different treatment quality based on underlying bleeding profiles in patients with VTE. The lower TTR could influence the effect estimates on major bleeding events in our study, resulting in a milder observed relative risk for bleeding. However, due to limited information on warfarin treatment quality in the HIRA database, further investigations are warranted.

Adherence to treatment in real-world practice is usually lower compared to clinical trials, where there are various methods to maximize adherence over the course of the trial. Given the poor adherence in routine clinical practice, we used the as-treated approach to assess the treatment effect in patients who continued their initial therapy. In contrast, RCTs generally adopt the intention-to-treat approach to estimate the effect of the initial assigned treatment, which yields conservative estimates, as it does not account for treatment switching or discontinuation throughout follow-up [39]. Another possible factor impacting treatment effect estimate could be discrepancies in the definitions of follow-up between RCTs and corresponding emulations, leading to differential follow-up durations where some significant outcomes could have occurred after switching treatment or discontinuation.

The incidence of VTE in Asians is lower than that in Caucasian populations [40], which may partly explain the observed differences between the RCT and emulation estimates. All selected trials, except for AMPLIFY, had subgroup results for Asians. The EINSTEIN-PE (4.1% vs. 2.1%) and RE-COVER II (2.4% vs. 1.0%) trials reported a higher proportion of the efficacy outcome with NOACs versus conventional treatment [7, 10], while the EINSTEIN-DVT (1.4% vs. 3.8%) and Hokusai-VTE (3.1% vs. 3.9%) trials showed results consistent with the main analyses [8, 9, 41]. However, the results of efficacy and safety outcomes in the Asian subgroup are limited by statistical power, and thus, further research is needed to fully explain the discrepancies between the estimates of RCTs and RWE studies.

This study has several limitations. First, exposure misclassification should be considered. Prescription records did not confirm whether the patient had actually administered the medication. However, the as-treated approach with a 10-day grace period can substantially complement this limitation. Additionally, a relatively short washout period used to define new users of oral anticoagulants may introduce the possibility of population misclassification. However, we believe that the look-back period of the 180 days provided a pragmatic approach to balance the trade-off between sample size and the potential of misclassification. This approach allowed us to secure a sufficiently large sample size, enhancing the statistical power of our study. Second, outcome misclassifications may have occurred. Using diagnostic codes cannot capture all recurrent VTE, and false positives are possible. Nevertheless, we optimized our outcome definition using previously validated algorithms, which had an 80–90% PPV. Moreover, while the HIRA database primarily captures in-hospital deaths, it may not fully capture information on deaths occurring at home or in other settings, potentially leading to outcome or follow-up misclassification due to censoring. However, it is important to note that according to national statistics in South Korea for the year 2022, approximately 74.8% of all deaths occurred in hospitals [42]. Additionally, considering that our study population consists of patients with venous thromboembolism (VTE) who may require ongoing healthcare services due to their vulnerability and treatment needs, the impact of outcome or follow-up misclassification is likely to be minimal in this study. Third, residual confounders potentially remain despite accounting for 69 covariates, as laboratory test results were unavailable in the HIRA database. Fourth, several inclusion and exclusion criteria, such as patient willingness, expectations, and laboratory tests, could not be emulated, as these were not captured in the HIRA database. In clinical practice, the use of such a proxy may affect replicability, because diagnostic test results do not directly lead to diagnosis. In short, the operational definition of emulating inclusion and exclusion criteria in this study could be slightly different from the intended meaning of RCT criteria. Lastly, we had a relatively short follow-up period and limited statistical power, despite the use of nationwide data [43]. South Korea has not reimbursed NOACs prescribed for more than 6 months until 2019, and this could result in different maximum follow-up times between NOAC and warfarin groups. However, since NOACs are recommended for 6 months in routine practices, this effect is unlikely to be significant in this study [11].

## Conclusions

We found that RWE studies, compared with their corresponding RCTs, can deduce similar conclusions in NOAC-VTE settings, suggesting the possible use of non-randomized RWE to complement RCTs for regulatory decision-making. Although the RCT-RWE agreement was not met for all binary metrics, the failure of RWE studies to meet metrics after emulation does not necessarily lead to questioning its reliability, as real-world practice is substantially different from well-controlled trial environments. Likewise, RWE studies satisfying all metrics do not preclude the possibility of chance findings and thus do not confirm validity. This is because prominent emulation differences and other biases can remain in non-randomized RWE studies, including residual confounding, misclassification of exposure or outcome, informative censoring, and adherence to treatment. Although RWE studies cannot completely substitute RCTs, they can act as suggestive data for regulatory decision-making, particularly in situations where RCTs are infeasible due to ethical or conflicting issues. Emulating RCTs based on routine clinical practice has subtle differences, and one of these may be driven by the differential baseline characteristics and treatment quality between populations in RCTs and RWE studies. Further studies using other sources of real-world data and on different treatments would benefit healthcare providers and regulatory authorities by increasing their confidence in the validity and reliability of RWE studies for future clinical and regulatory decision-making.

### Supplementary Information

**Additional file 1:** **Table S1. **Codes used to identify the effectiveness outcome: Recurrent VTE. **Table S2. **Definitions of the potential confounders. **Table S3. **Codes used to identify the safety outcome: Major bleeding. **Table S4.** Constructing the emulation cohort with the same criteria of the AMPLIFY trial. **Table S5. **Constructing the emulation cohort with the same criteria of the RE-COVER II trial. **Table S6. **Constructing the emulation cohort with the same criteria of the Hokusai-VTE trial. **Table S7. **Constructing the emulation cohort with the same criteria of the EINSTEIN-DVT trial. **Table S8. **Constructing the emulation cohort with the same criteria of the EINSTEIN-PE trial. **Table S9. **Baseline characteristics of AMPLIFY emulation cohort. **Table S10. **Baseline characteristics of RE-COVER II emulation cohort. **Table S11. **Baseline characteristics of Hokusai-VTE emulation cohort. **Table S12. **Baseline characteristics of EINSTEIN-DVT emulation cohort. **Table S13. **Baseline characteristics of EINSTEIN-PE emulation cohort. **Table S14. **Sensitivity analyses of intention-to-treat approach on the effectiveness and safety of RWE emulation study. **Table S15. **Sensitivity analyses of asymmetrical trimming and adjusting for PS deciles on the effectiveness and safety of RWE emulation study. **Fig. S1. **Design diagram of inclusion and exclusion criteria in AMPLIFY pivotal trial emulation. **Fig. S2. **Design diagram of inclusion and exclusion criteria in RE-COVER II pivotal trial emulation. **Fig. S3. **Design diagram of inclusion and exclusion criteria in Hokusai-VTE pivotal trial emulation. **Fig. S4. **Design diagram of inclusion and exclusion criteria in EINSTEIN-DVT pivotal trial emulation. **Fig. S5. **Design diagram of inclusion and exclusion criteria in EINSTEIN-PE pivotal trial emulation. **Fig. S6. **Distributions of PS in the unmatched and matched cohort in AMPLIFY emulation study. **Fig. S7. **Distributions of PS in the unmatched and matched cohort in RE-COVER II emulation study. **Fig. S8. **Distributions of PS in the unmatched and matched cohort in Hokusai-VTE emulation study. **Fig. S9. **Distributions of PS in the unmatched and matched cohort in EINSTEIN-DVT emulation study. **Fig. S10. **Distributions of PS in the unmatched and matched cohort in EINSTEIN-PE emulation study.

## Data Availability

Detailed study design and protocols for each emulation are available on the Real-World Evidence Registry, which is part of the Real-World Evidence Transparency Initiative (links to public registration: https://osf.io/9whp5 [AMPLIFY], https://osf.io/ewfv4 [RE-COVER II], https://osf.io/z5ha2 [Hokusai-VTE], https://osf.io/y9kxw [EINSTEIN-DVT], https://osf.io/zw89j [EINSTEIN-PE]). The data supporting the findings of this study are available from the Health Insurance Review and Assessment Service of South Korea. However, access to these data is restricted by domestic laws and regulations that prohibit the public distribution or release of individual data. Consequently, the data are not publicly available. Interested parties can obtain the data from the authors upon making a reasonable request and obtaining permission from the Health Insurance Review and Assessment Service of South Korea.

## Abbreviations

AMPLIFY: Apixaban for the Initial Management of Pulmonary Embolism and Deep-Vein Thrombosis as First-Line Therapy
CI: Confidence interval
DVT: Deep vein thrombosis
EA: Estimate agreement
ED: Emergency department
EINSTEIN-DVT: Oral Direct Factor Xa Inhibitor Rivaroxaban in Patients With Acute Symptomatic Deep Vein Thrombosis
EINSTEIN-PE: Oral Direct Factor Xa Inhibitor Rivaroxaban in Patients With Acute Symptomatic Pulmonary Embolism
HIRA: Health Insurance Review and Assessment
Hokusai-VTE: Comparative Investigation of Low Molecular Weight Heparin/Edoxaban Tosylate Versus Heparin/Warfarin in the Treatment of Symptomatic Deep-Vein Blood Clots and/or Lung Blood Clots
HR: Hazard ratio
ICD: International classification of diseases
ITT: Intention-to-treat
NOACs: Non-vitamin K antagonist oral anticoagulants
PE: Pulmonary embolism
PPV: Positive predictive value
PS: Propensity score
RA: Regulatory agreement
RCT: Randomized controlled trial
RCT DUPLICATE: Randomized Controlled Trials Duplicated Using Prospective Longitudinal Insurance Claims: Applying Techniques of Epidemiology
RE-COVER II: Phase III Study Testing Efficacy & Safety of Oral Dabigatran Etexilate vs. Warfarin for 6-month Treatment for Acute Symptomatic Venous Thromboembolism
RR: Relative risk
RWE: Real-world evidence
SD: Standardized difference
TTR: Time in therapeutic range
VTE: Venous thromboembolism

## References

[1] Franklin JM, Schneeweiss S (2017). When and how can real world data analyses substitute for randomized controlled trials?. Clin Pharmacol Ther.

[2] Hernán MA, Robins JM (2016). Using big data to emulate a target trial when a randomized trial is not available. Am J Epidemiol.

[3] Sherman RE, Anderson SA, Dal Pan GJ, Gray GW, Gross T, Hunter NL, LaVange L, Marinac-Dabic D, Marks PW, Robb MA (2016). Real-world evidence - what is it and what can it tell us?. N Engl J Med.

[4] Jarow JP, LaVange L, Woodcock J (2017). Multidimensional evidence generation and FDA regulatory decision making: defining and using “real-world” data. JAMA.

[5] Franklin JM, Patorno E, Desai RJ, Glynn RJ, Martin D, Quinto K, Pawar A, Bessette LG, Lee H, Garry EM (2021). Emulating randomized clinical trials with nonrandomized real-world evidence studies: first results from the RCT DUPLICATE Initiative. Circulation.

[6] Agnelli G, Buller HR, Cohen A, Curto M, Gallus AS, Johnson M, Masiukiewicz U, Pak R, Thompson J, Raskob GE (2013). Oral apixaban for the treatment of acute venous thromboembolism. N Engl J Med.

[7] Schulman S, Kakkar AK, Goldhaber SZ, Schellong S, Eriksson H, Mismetti P, Christiansen AV, Friedman J, Le Maulf F, Peter N (2014). Treatment of acute venous thromboembolism with dabigatran or warfarin and pooled analysis. Circulation.

[8] Büller HR, Décousus H, Grosso MA, Mercuri M, Middeldorp S, Prins MH, Raskob GE, Schellong SM, Schwocho L, Segers A (2013). Edoxaban versus warfarin for the treatment of symptomatic venous thromboembolism. N Engl J Med.

[9] Bauersachs R, Berkowitz SD, Brenner B, Buller HR, Decousus H, Gallus AS, Lensing AW, Misselwitz F, Prins MH, Raskob GE (2010). Oral rivaroxaban for symptomatic venous thromboembolism. N Engl J Med.

[10] Büller HR, Prins MH, Lensin AW, Decousus H, Jacobson BF, Minar E, Chlumsky J, Verhamme P, Wells P, Agnelli G (2012). Oral rivaroxaban for the treatment of symptomatic pulmonary embolism. N Engl J Med.

[11] Ortel TL, Neumann I, Ageno W, Beyth R, Clark NP, Cuker A, Hutten BA, Jaff MR, Manja V, Schulman S (2020). American Society of Hematology 2020 guidelines for management of venous thromboembolism: treatment of deep vein thrombosis and pulmonary embolism. Blood Adv.

[12] Kim JA, Yoon S, Kim LY, Kim DS (2017). Towards actualizing the value potential of Korea Health Insurance Review and Assessment (HIRA) data as a resource for health research: strengths, limitations, applications, and strategies for optimal use of HIRA data. J Korean Med Sci.

[13] Alotaibi GS, Wu C, Senthilselvan A, McMurtry MS (2015). The validity of ICD codes coupled with imaging procedure codes for identifying acute venous thromboembolism using administrative data. Vasc Med.

[14] Albertsen IE, Nielsen PB (2020). How to optimize the value of administrative venous thromboembolism codes. Thromb Res.

[15] Lee AY, Levine MN, Baker RI, Bowden C, Kakkar AK, Prins M, Rickles FR, Julian JA, Haley S, Kovacs MJ (2003). Low-molecular-weight heparin versus a coumarin for the prevention of recurrent venous thromboembolism in patients with cancer. N Engl J Med.

[16] Kim HY, Chang SA, Kim KH, Kim JY, Seo WK, Kim H, Seo JS, Shin SH, Rhee SJ, Lee SH (2021). Epidemiology of venous thromboembolism and treatment pattern of oral anticoagulation in Korea, 2009–2016: a nationwide study based on the National Health Insurance Service Database. J Cardiovasc Imaging.

[17] Petri H, Urquhart J (1991). Channeling bias in the interpretation of drug effects. Stat Med.

[18] Rassen JA, Shelat AA, Myers J, Glynn RJ, Rothman KJ, Schneeweiss S (2012). One-to-many propensity score matching in cohort studies. Pharmacoepidemiol Drug Saf.

[19] Park J, Kwon S, Choi E-K, Choi Y-J, Lee E, Choe W, Lee S-R, Cha M-J, Lim W-H, Oh S (2019). Validation of diagnostic codes of major clinical outcomes in a National Health Insurance database. Int J Arrhythmia.

[20] Hernán MA, Hernández-Díaz S (2012). Beyond the intention-to-treat in comparative effectiveness research. Clin Trials.

[21] Murray EJ, Caniglia EC, Petito LC (2021). Causal survival analysis: a guide to estimating intention-to-treat and per-protocol effects from randomized clinical trials with non-adherence. Res Methods Med Health Sci.

[22] Stürmer T, Rothman KJ, Avorn J, Glynn RJ (2010). Treatment effects in the presence of unmeasured confounding: dealing with observations in the tails of the propensity score distribution–a simulation study. Am J Epidemiol.

[23] Desai RJ, Franklin JM (2019). Alternative approaches for confounding adjustment in observational studies using weighting based on the propensity score: a primer for practitioners. BMJ.

[24] Franklin JM, Pawar A, Martin D, Glynn RJ, Levenson M, Temple R, Schneeweiss S (2020). Nonrandomized real-world evidence to support regulatory decision making: process for a randomized trial replication project. Clin Pharmacol Ther.

[25] Wang SV, Schneeweiss S, Franklin JM, Desai RJ, Feldman W, Garry EM, Glynn RJ, Lin KJ, Paik J, Patorno E (2023). Emulation of randomized clinical trials with nonrandomized database analyses: results of 32 clinical trials. JAMA.

[26] Franklin JM, Glynn RJ, Suissa S, Schneeweiss S (2020). Emulation differences vs. biases when calibrating real-world evidence findings against randomized controlled trials. Clin Pharmacol Ther.

[27] Dentali F, Sironi AP, Gianni M, Orlandini F, Guasti L, Grandi AM, Franchini M, Ageno W, Squizzato A (2015). Gender difference in efficacy and safety of nonvitamin K antagonist oral anticoagulants in patients with nonvalvular atrial fibrillation or venous thromboembolism: a systematic review and a meta-analysis of the literature. Semin Thromb Hemost.

[28] Loffredo L, Violi F, Perri L (2016). Sex related differences in patients with acute venous thromboembolism treated with new oral anticoagulants. A meta-analysis of the interventional trials. Int J Cardiol.

[29] Palareti G, Legnani C, Antonucci E, Cosmi B, Falanga A, Poli D, Mastroiacovo D, Pengo V, Ageno W, Testa S (2021). Do women with venous thromboembolism bleed more than men during anticoagulation? Data from the real-life, prospective START-Register. Ther Adv Drug Saf.

[30] Lapner S, Cohen N, Kearon C (2014). Influence of sex on risk of bleeding in anticoagulated patients: a systematic review and meta-analysis. J Thromb Haemost.

[31] Engbers MJ, van Hylckama VA, Rosendaal FR (2010). Venous thrombosis in the elderly: incidence, risk factors and risk groups. J Thromb Haemost.

[32] Geldhof V, Vandenbriele C, Verhamme P, Vanassche T (2014). Venous thromboembolism in the elderly: efficacy and safety of non-VKA oral anticoagulants. Thromb J.

[33] Tritschler T, Castellucci LA, Van Es N, Aujesky D, Le Gal G (2020). Treatment of venous thromboembolism in elderly patients in the era of direct oral anticoagulants. Pol Arch Intern Med.

[34] Larsen TB, Nielsen PB, Skjøth F, Rasmussen LH, Lip GY (2014). Non-vitamin K antagonist oral anticoagulants and the treatment of venous thromboembolism in cancer patients: a semi systematic review and meta-analysis of safety and efficacy outcomes. PLoS One.

[35] VanderWeele TJ, Robins JM (2007). Four types of effect modification: a classification based on directed acyclic graphs. Epidemiology.

[36] Ma C (2012). Current antithrombotic treatment in East Asia: some perspectives on anticoagulation and antiplatelet therapy. Thromb Haemost.

[37] Cho H, Kang J, Kim HS, Park KW (2020). Ethnic differences in oral antithrombotic therapy. Korean Circ J.

[38] Chiang CE, Wang KL, Lip GY (2014). Stroke prevention in atrial fibrillation: an Asian perspective. Thromb Haemost.

[39] McCoy CE (2017). Understanding the intention-to-treat principle in randomized controlled trials. West J Emerg Med.

[40] Cohen A, Chiu KM, Park K, Jeyaindran S, Tambunan KL, Ward C, Wong R, Yoon SS (2012). Managing venous thromboembolism in Asia: winds of change in the era of new oral anticoagulants. Thromb Res.

[41] Lee YJ (2014). Use of novel oral anticoagulants for the treatment of venous thromboembolism and its considerations in Asian patients. Ther Clin Risk Manag.

[42] Preliminary results of birth and death statistics in 2022. https://kostat.go.kr/board.es?mid=a20108010000&bid=11773&act=view&list_no=424347&tag=&nPage=1&ref_bid=11742,11743,11744,11745,11746,11747,11748,11749,11773,11774,11750.

[43] Jones SR, Carley S, Harrison M (2003). An introduction to power and sample size estimation. Emerg Med J.

