# Supplementary material for: Real-world data emulating randomized controlled trials of non-vitamin K antagonist oral anticoagulants in patients with venous thromboembolism
Source: BMC Med. 2023 Sep 29;21:375. doi: 10.1186/s12916-023-03069-1 (PMC10542685; doi:10.1186/s12916-023-03069-1)
Supplement: Supplementary file 1 — Additional file 1: Table S1. Codes used to identify the effectiveness outcome: Recurrent VTE. Table S2. Definitions of the potential confounders. Table S3. Codes used to identify the safety outcome: Major bleeding. Table S4. Constructing the emulation cohort with the same criteria of the AMPLIFY trial. Table S5. Constructing the emulation cohort with the same criteria of the RE-COVER II trial. Table S6. Constructing the emulation cohort with the same criteria of the Hokusai-VTE trial. Table S7. Constructing the emulation cohort with the same criteria of the EINSTEIN-DVT trial. Table S8. Constructing the emulation cohort with the same criteria of the EINSTEIN-PE trial. Table S9. Baseline characteristics of AMPLIFY emulation cohort. Table S10. Baseline characteristics of RE-COVER II emulation cohort. Table S11. Baseline characteristics of Hokusai-VTE emulation cohort. Table S12. Baseline characteristics of EINSTEIN-DVT emulation cohort. Table S13. Baseline characteristics of EINSTEIN-PE emulation cohort. Table S14. Sensitivity analyses of intention-to-treat approach on the effectiveness and safety of RWE emulation study. Table S15. Sensitivity analyses of asymmetrical trimming and adjusting for PS deciles on the effectiveness and safety of RWE emulation study. Fig. S1. Design diagram of inclusion and exclusion criteria in AMPLIFY pivotal trial emulation. Fig. S2. Design diagram of inclusion and exclusion criteria in RE-COVER II pivotal trial emulation. Fig. S3. Design diagram of inclusion and exclusion criteria in Hokusai-VTE pivotal trial emulation. Fig. S4. Design diagram of inclusion and exclusion criteria in EINSTEIN-DVT pivotal trial emulation. Fig. S5. Design diagram of inclusion and exclusion criteria in EINSTEIN-PE pivotal trial emulation. Fig. S6. Distributions of PS in the unmatched and matched cohort in AMPLIFY emulation study. Fig. S7. Distributions of PS in the unmatched and matched cohort in RE-COVER II emulation study. Fig. S8. Distributions of PS in th [file 12916_2023_3069_MOESM1_ESM.docx]

**Additional file**

This additional file has been provided by the authors to provide readers with further information about their work.

**Supplemental Table Legends**

Table S1. Codes used to identify the effectiveness outcome: Recurrent VTE 13

Table S2. Definitions of the potential confounders 14

Table S3. Codes used to identify the safety outcome: Major bleeding 19

Table S4. Constructing the emulation cohort with the same criteria of the AMPLIFY trial 20

Table S5. Constructing the emulation cohort with the same criteria of the RE-COVER II trial 21

Table S6. Constructing the emulation cohort with the same criteria of the Hokusai-VTE trial 22

Table S7. Constructing the emulation cohort with the same criteria of the EINSTEIN-DVT trial 23

Table S8. Constructing the emulation cohort with the same criteria of the EINSTEIN-PE trial 24

Table S9. Baseline characteristics of AMPLIFY emulation cohort 25

Table S10. Baseline characteristics of RE-COVER II emulation cohort 28

Table S11. Baseline characteristics of Hokusai-VTE emulation cohort 31

Table S12. Baseline characteristics of EINSTEIN-DVT emulation cohort 34

Table S13. Baseline characteristics of EINSTEIN-PE emulation cohort 37

Table S14. Sensitivity analyses of intention-to-treat approach on the effectiveness and safety of RWE emulation study 40

Table S15. Sensitivity analyses of asymmetrical trimming and adjusting for PS deciles on the effectiveness and safety of RWE emulation study 41

**Supplemental Figure Legends**

[Fig S1. Design diagram of inclusion and exclusion criteria in AMPLIFY pivotal trial emulation 3](#_Toc144371355)

[Fig S2. Design diagram of inclusion and exclusion criteria in RE-COVER II pivotal trial emulation 4](#_Toc144371356)

[Fig S3. Design diagram of inclusion and exclusion criteria in Hokusai-VTE pivotal trial emulation 5](#_Toc144371357)

[Fig S4. Design diagram of inclusion and exclusion criteria in EINSTEIN-DVT pivotal trial emulation 6](#_Toc144371358)

[Fig S5. Design diagram of inclusion and exclusion criteria in EINSTEIN-PE pivotal trial emulation 7](#_Toc144371359)

[Fig S6. Distributions of PS in the unmatched and matched cohort in AMPLIFY emulation study 8](#_Toc144371360)

[Fig S7. Distributions of PS in the unmatched and matched cohort in RE-COVER II emulation study 9](#_Toc144371361)

[Fig S8. Distributions of PS in the unmatched and matched cohort in Hokusai-VTE emulation study 10](#_Toc144371362)

[Fig S9. Distributions of PS in the unmatched and matched cohort in EINSTEIN-DVT emulation study 11](#_Toc144371363)

[Fig S10. Distributions of PS in the unmatched and matched cohort in EINSTEIN-PE emulation study 12](#_Toc144371364)


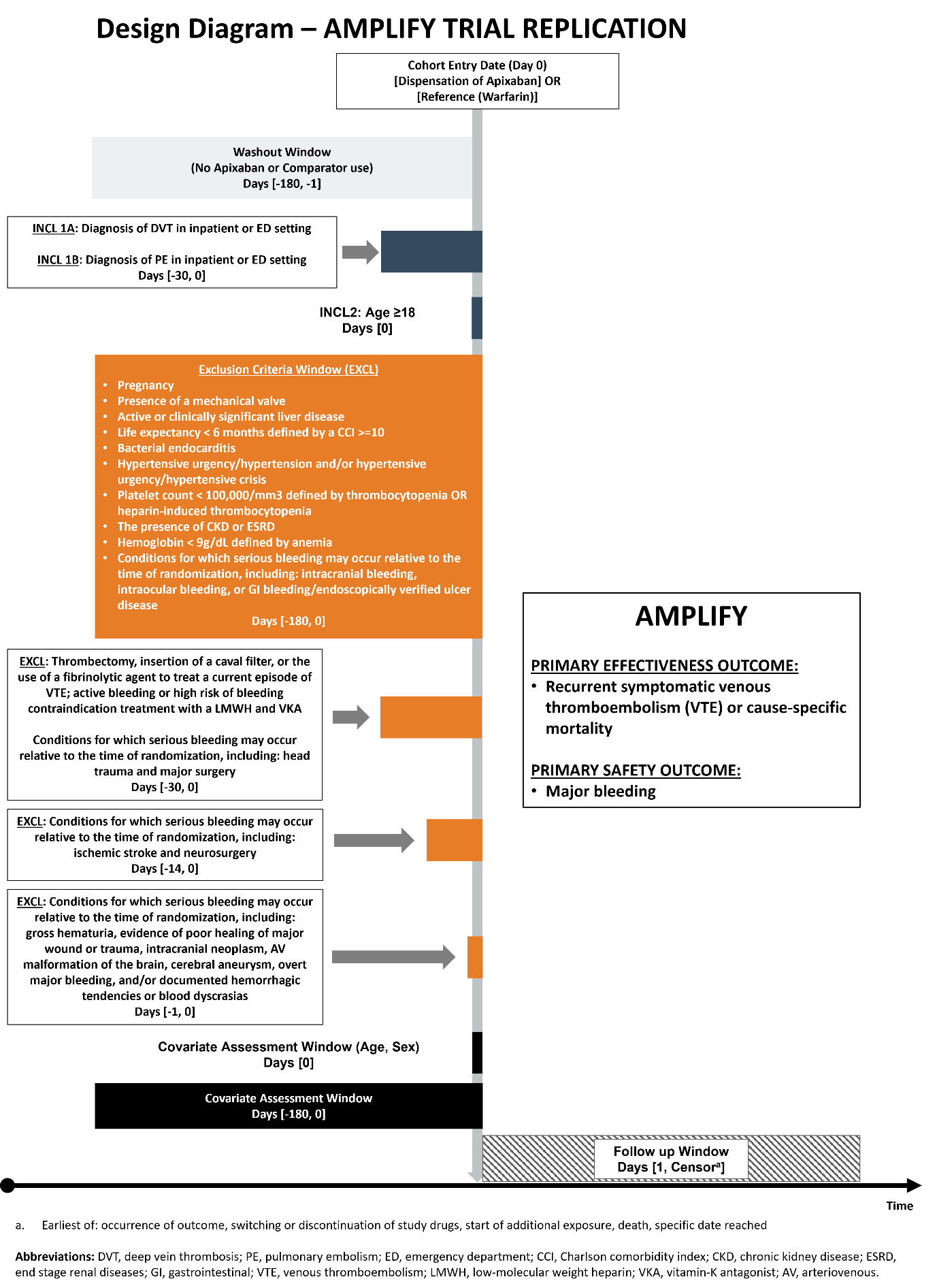


Fig S1. Design diagram of inclusion and exclusion criteria in AMPLIFY pivotal trial emulation


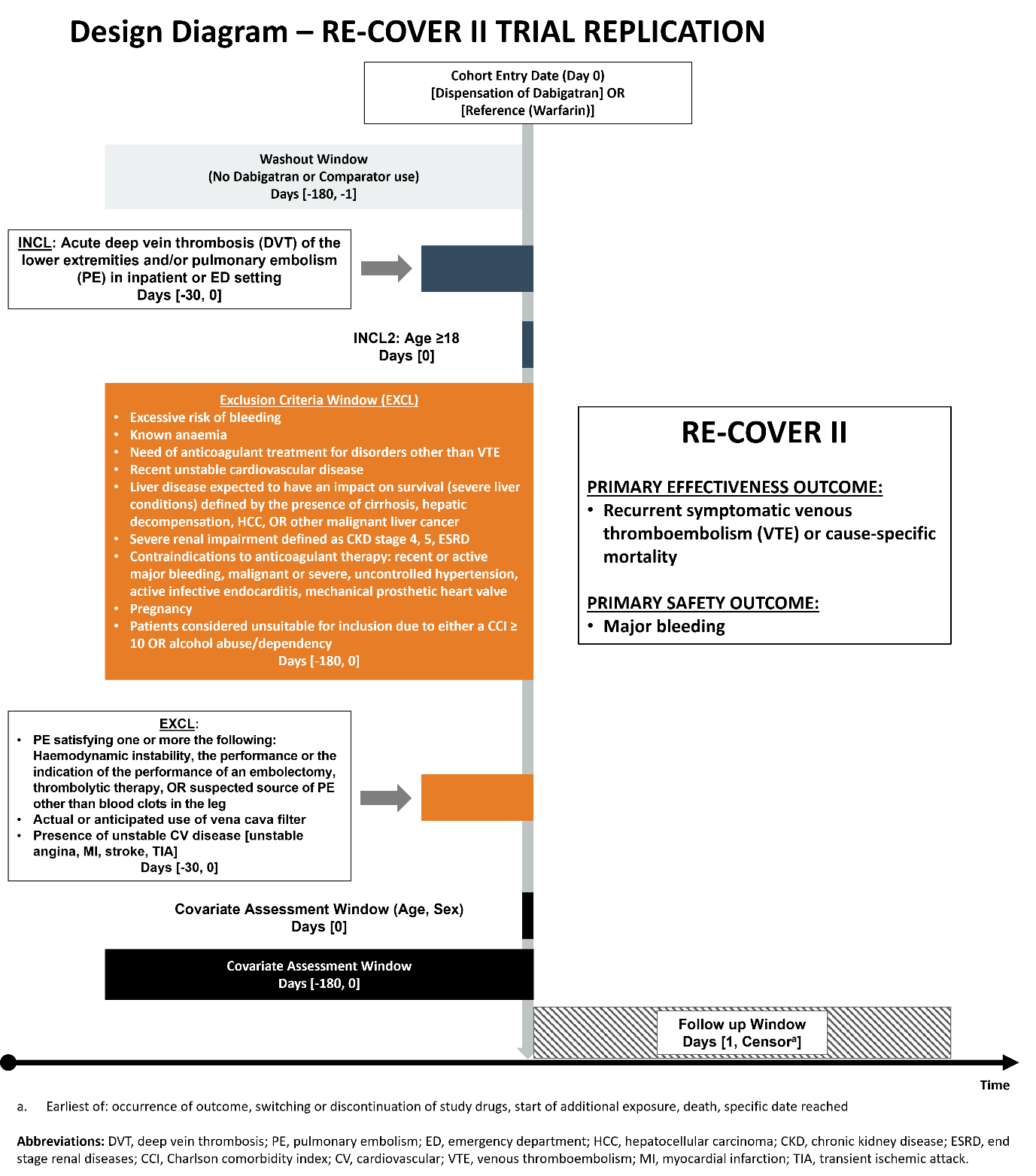


Fig S2. Design diagram of inclusion and exclusion criteria in RE-COVER II pivotal trial emulation


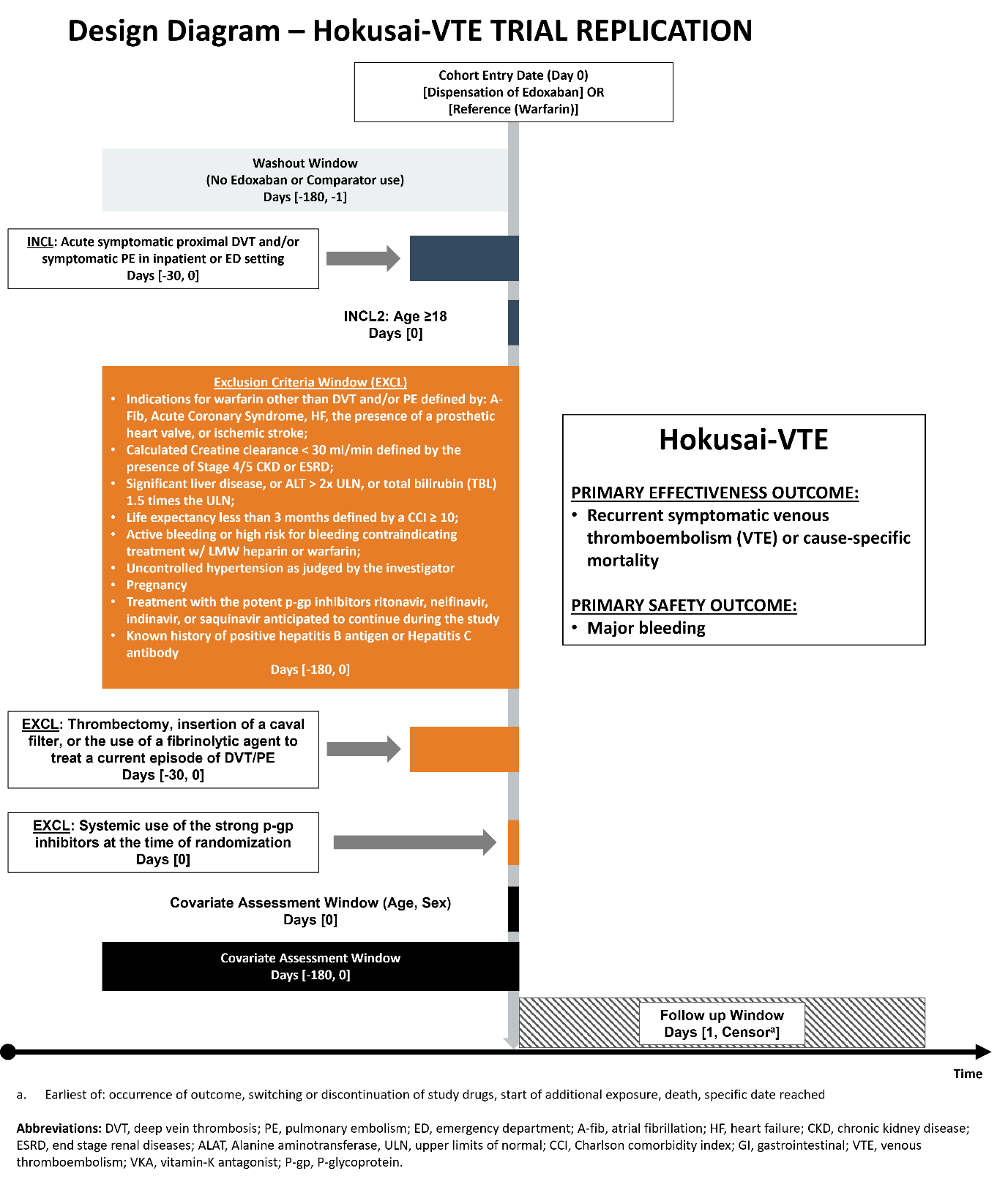


Fig S3. Design diagram of inclusion and exclusion criteria in Hokusai-VTE pivotal trial emulation


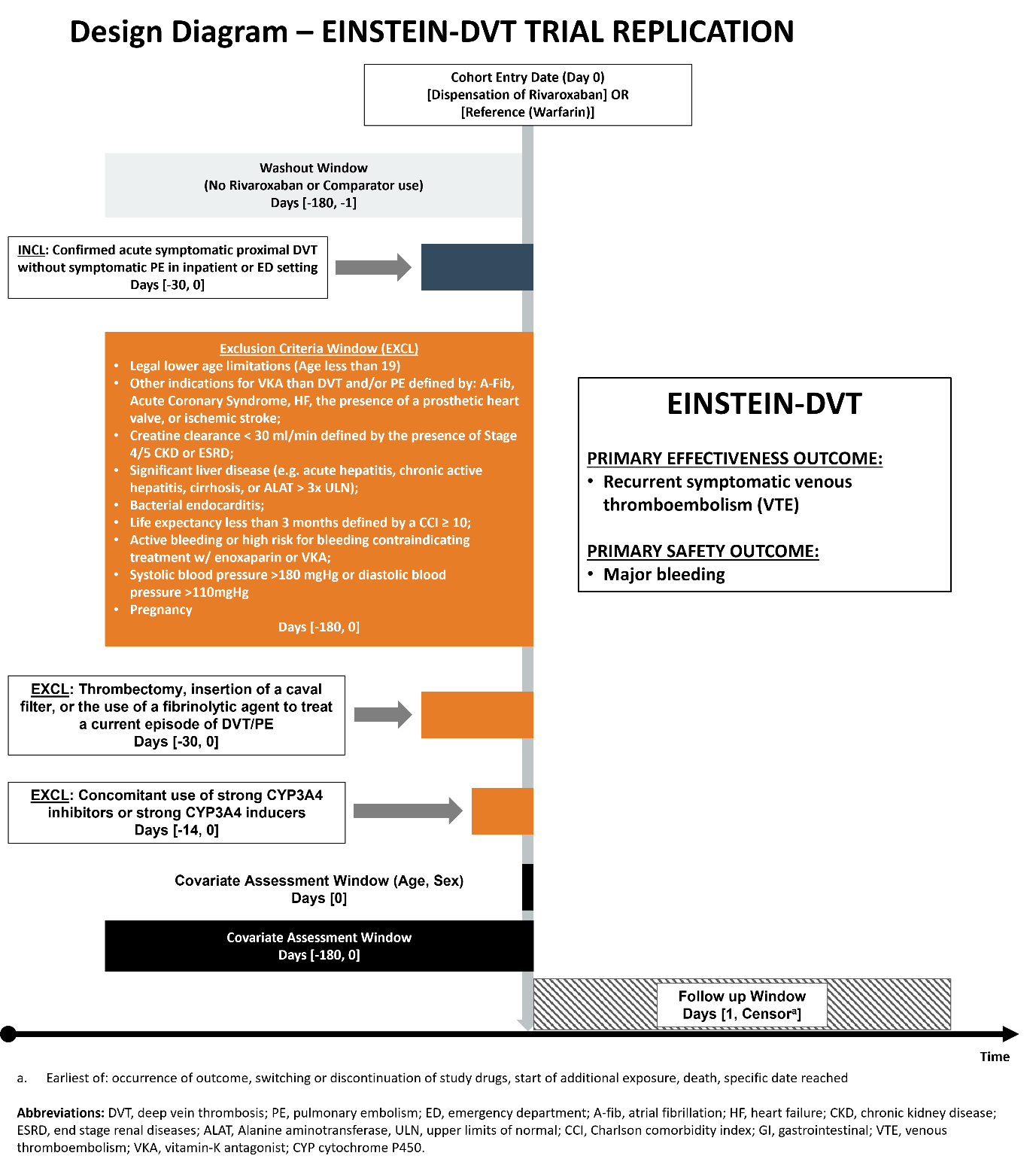


Fig S4. Design diagram of inclusion and exclusion criteria in EINSTEIN-DVT pivotal trial emulation


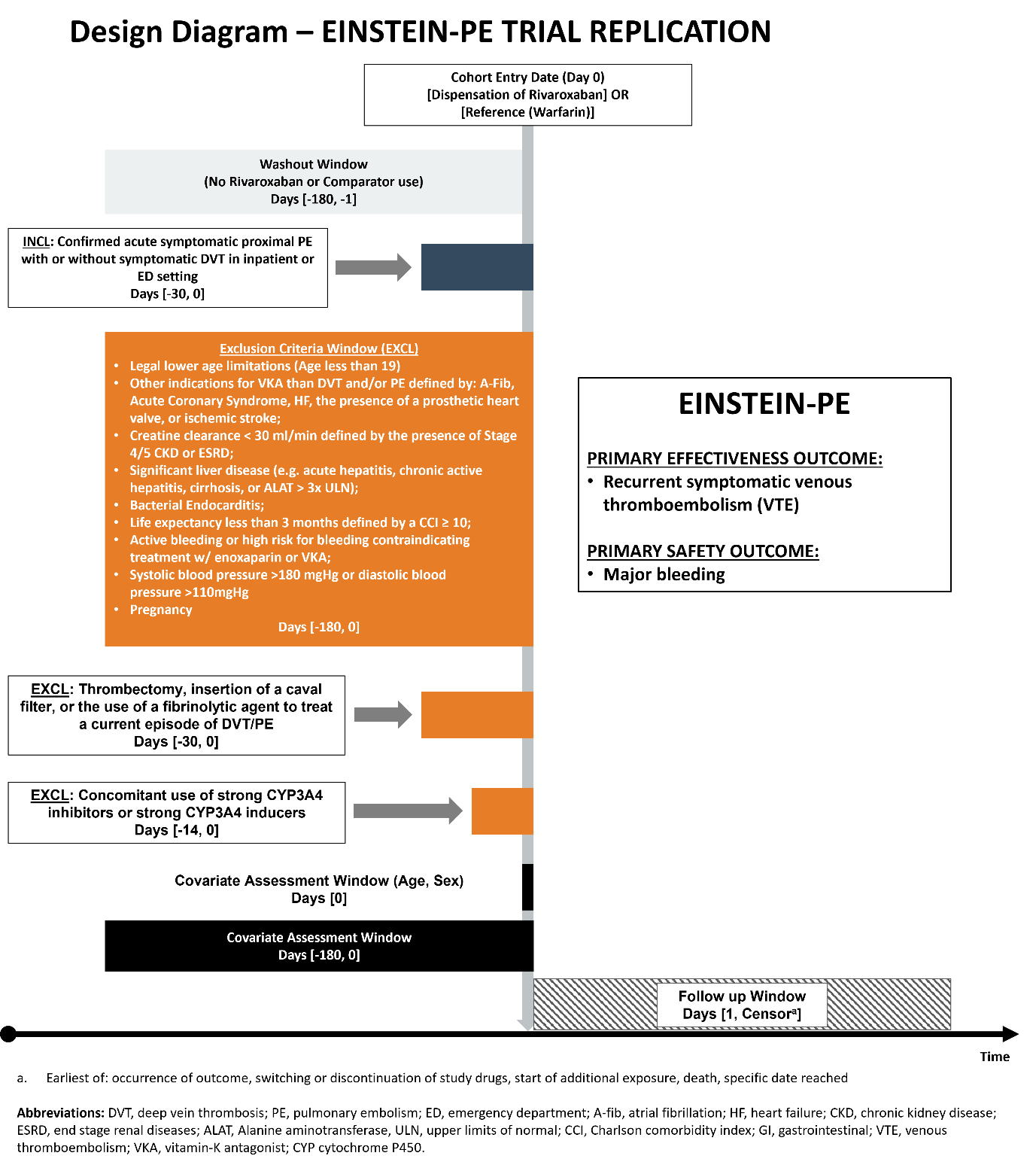


Fig S5. Design diagram of inclusion and exclusion criteria in EINSTEIN-PE pivotal trial emulation


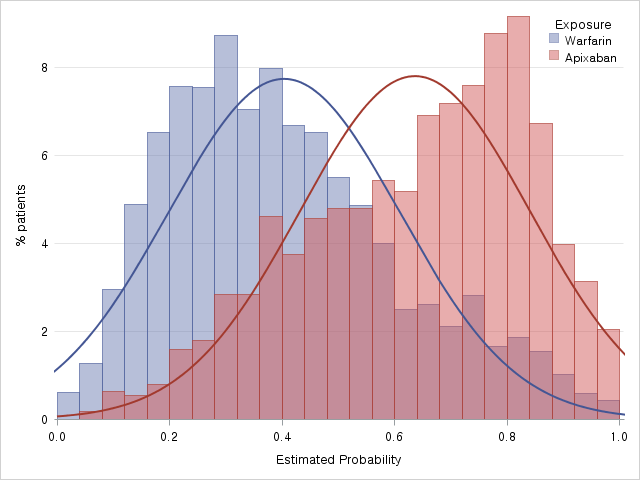

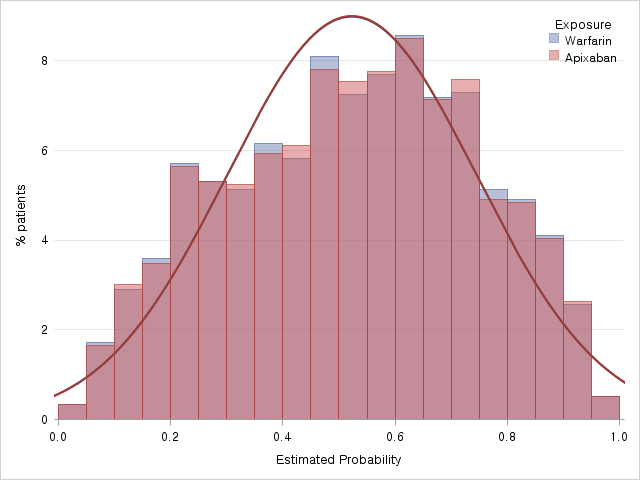


Fig S6. Distributions of PS in the unmatched and matched cohort in AMPLIFY emulation study


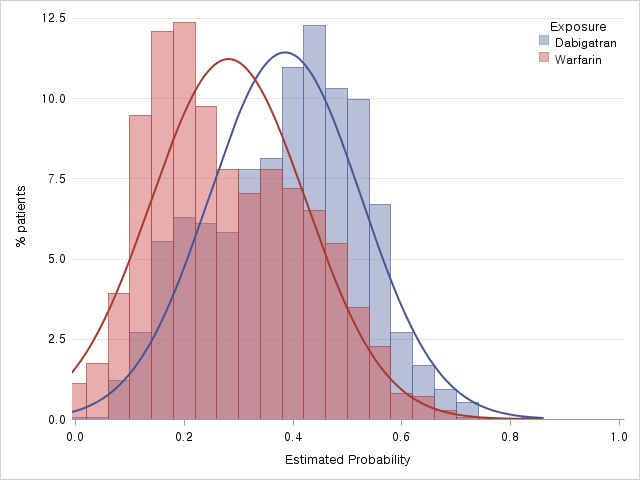

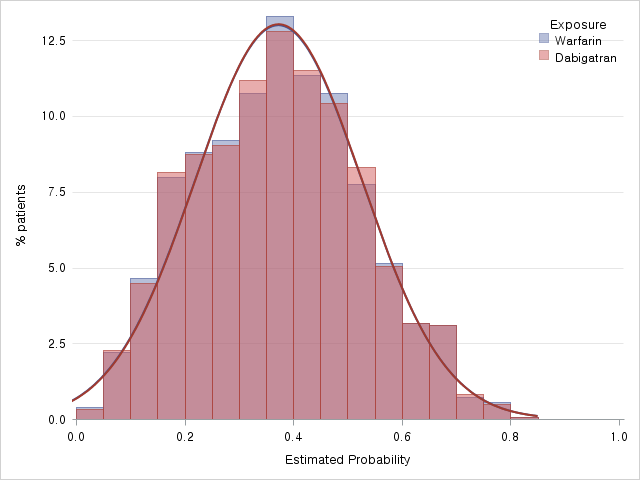


Fig S7. Distributions of PS in the unmatched and matched cohort in RE-COVER II emulation study


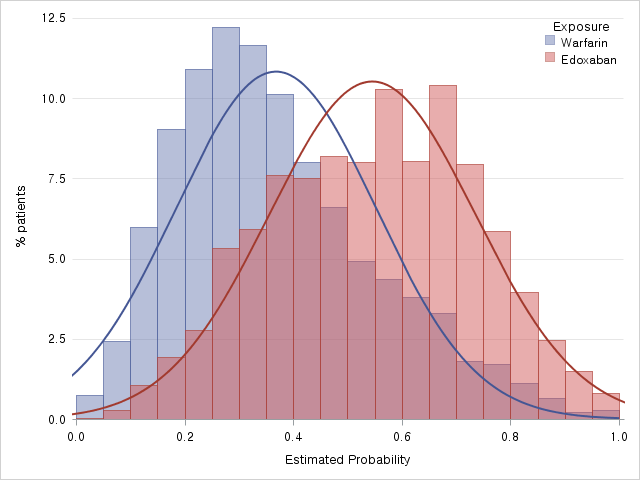

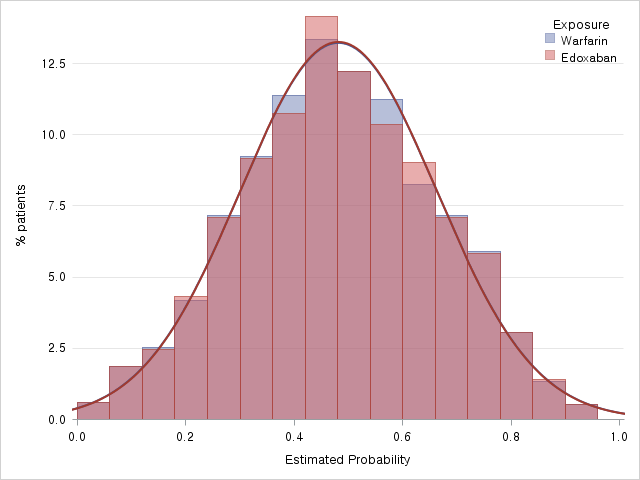


Fig S8. Distributions of PS in the unmatched and matched cohort in Hokusai-VTE emulation study


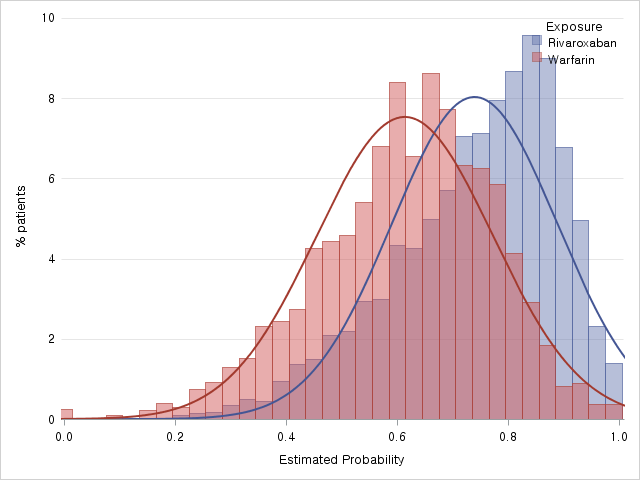

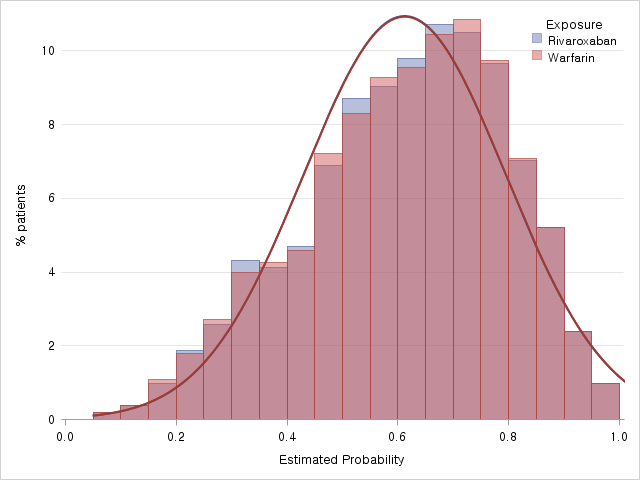


Fig S9. Distributions of PS in the unmatched and matched cohort in EINSTEIN-DVT emulation study


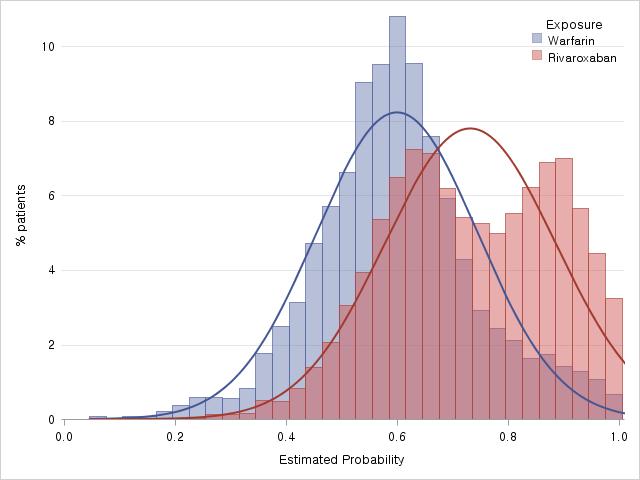

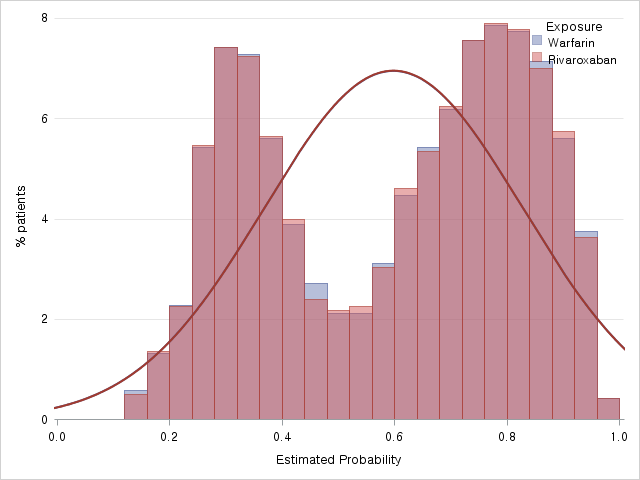


Fig S10. Distributions of PS in the unmatched and matched cohort in EINSTEIN-PE emulation study

Table S1. Codes used to identify the effectiveness outcome: Recurrent VTE

| **Outcome** | **Setting** | **Description** | **ICD-10 code or procedure code** |
| --- | --- | --- | --- |
| Deep vein thrombosis | Primary diagnosis + Hospitalization | Phlebitis and thrombophlebitis | I801, I802, I803 |
|  |  | Other venous embolism and thrombosis | I822, I828, I829 |
| Pulmonary embolism | Primary diagnosis + Hospitalization | Pulmonary embolism with or without mention of acute cor pulmonale | I260, I269 |
| Procedure | | Doppler ultrasonography | EB484, EB485, EB486, EB487, EB488, EB489 |
|  |  | Computer tomography scan | HA457, HA467, HA477, HA447, HA458, HA468, HA478, HA448, HA424, HA434, HA464, HA474, HA444, HA851, HA801, HA805, HA809, HA813, HA853, HA834, HA835, HA836, HA856, HA857, HA858, HA859 |
|  |  | Venography | HA711, HA712, HA713, HA714, HA731, HA732, HA741, HA742, HA743, HC241, HC242 |
| VTE-related death | Primary diagnosis | Claims of diagnosis with VTE, where patient status on the end date of care was recorded as ‘death.’ | |

**Abbreviations:** ICD-10, International Classification of Diseases 10^th^ revision

Table S2. Definitions of the potential confounders

| **Categories** | **Definition/codes** |
| --- | --- |
| **Comorbidity conditions** | **ICD-10 codes** |
| Previous VTE | I80, I82, I26 |
| Active cancer | Defined as the first diagnosis of cancer (C00-C97, except C44) with V code (V011, V027, V193, V194); or receiving anti-cancer treatment (chemotherapy, radiation therapy, or cancer-related surgery) under cancer diagnosis with V code  Chemotherapy: L01-04, H02AB02, H02AB04, H02AB06, H02AB07, H02AB09  Radiation therapy: HD051-HD059, HD061, HD071-HD073, HD080-HD089, HD091-HD093, HD110-HD115, HD121, HD150, HD160, HD211, HD212, HZ271  Cancer-related surgery: N0232, N0284-N0286, NA281-NA284, N0335, N0404, N0405, N0435-N0437, N0940, O0961-O0963, O1045, O1047, O1048, O1224-O1227, O1251, O1252, O1401, O1403-O1405, O1410, O1421-O1424, O1431, O1432, O1471, O1484, O1486, O1572, O1592, O1596, O1982, P2091, P2093, P2123, P2124, Q2150, Q2181-Q2183, Q2203, Q2206, Q2232, Q2292-Q2294, Q2346, Q2347, Q2348, Q2361, Q2362, Q2363, Q2365-Q2369, Q2401, Q2402, Q2403, Q2502, QA536, Q2533, Q2534, Q2536, Q2537, Q0251-Q0259, Q2594, Q2598, Q2601, Q2650, Q2651, QA671-QA673, QA679, Q1261, Q1262, Q2671-Q2673, Q2679, Q2761, Q2762, Q2791-Q2794, Q2796-Q2798, Q2890-Q2893, Q2901, Q0292, Q2921-Q2924, Q2927, Q2928, QA921-QA924, QA928, Q2925, Q2926, QA925, QA926, R3271, R3273, R3274, R3275, R3290, R3305, R3307, R3299, R3300, R3309, R3310, R3432, R3433, R3451, R3470, R3481, R3482, R3541-R3543, R3530, R3590, R3755, R3756, R3791, R3792, R3801, R3802, R3851, R3853, R3861, R3862, R3891, R3901, R3902, R3950, RZ512, R3960, R3975, R4003, R4004, R4067, R4068, R4071-R4074, R4140, R4143, R4144, R4147-R4149, R0141, R0142, R4154, R4155, R4156, R4250, R4331, R4332, R4423-R4428, P4543, P4551-P4554, P4561, P4571, P4572, S4616, S4634-S4639, S6691-6696, S4694-S4696, S4707-S4709, S4743, S4756-S4758, S4780, S4801-S4803, S4880, S4900, S4950, S5200, S5220, S5231, S5232, S5246, S5592, S5745, M6774, M6870, M6880, M6775, QZ841, M6890, M6900, M6991, M6910, Q0841, Q0842, N7136-N7139, N7140-N7153, Q7221-Q7225, Q7230, Q7280-Q7285, Q7342, Q7380, Q7410, Q7561-Q7567, Q7571, Q7572, Q7651-Q7654, Q7701-Q7703, QX706, Q7751, Q7752, Q7761-Q7763,Q7766, Q7771, Q7772, Q7775, Q7788, Q7789, U4811, U4812, U4881-U4883  Others: X5131-X5137 |
| Hemorrhagic stroke or other cerebrovascular disease | I60-I69 |
| Major trauma potentially causing prolonged immobilization | S020, S021, S04, S071, V00-V99, T08, T09, T10, T11, T12, T13, T14 |
| Chronic kidney disease Stage 1-2 | N181, N182 |
| Chronic kidney disease Stage 3-6 | N183-N185 |
| Acute kidney injury | N17 |
| Bladder stones or kidney stones | K800-K802, N20 |
| Alcohol abuse or dependence or drug abuse or dependence | F100, F101, F102, F103, T36, T37, T38, T39, T40, T41, T42, T43, T44, T45, T46, T47, T48, T49, T50 |
| Other atherosclerosis or cardiac conduction disorders, or Other CVD | I70, I45, I20, I21, I22, I23, I24, I25, I28, I42, I43, I50, I51, I52 |
| Diabetes with complication | E100, E101, E102, E103, E104, E105, E106, E107, E108, E110, E111, E112, E113, E114, E115, E116, E117, E118, E120, E121, E122, E123, E124, E125, E126, E127, E128, E130, E131, E132, E133, E134, E135, E136, E137, E138, E140, E141, E142, E143, E144, E145, E146, E147, E148 |
| Chronic kidney disease stage 3-6 + dialysis | N183-N185 |
| Systemic embolism | I744 |
| Coagulation defects | D65, D66, D67, D68, D69 |
| Diabetes: 1 inpatient or 2 outpatient claims within 180 days | E10-E14 |
| Intracranial or retroperitoneal hemorrhage: 1 inpatient or 2 outpatient claims within 180 days | I600, I601, I602, I603, I604, I605, I606, I607, I608, I609, I610, I611, I612, I613, I614, I615, I616, I618, I619, I620, I621, I629, K250, K252, K254, K256, K260, K262, K264, K266, K270, K272, K274, K276, K280, K282, K284, K286, K5521, K566, K5701, K5711, K5713, K5721, K5731, K5733, K5741, K5751, K5753, K5781, K5791, K5793, K625, K631, K661, K920, K921, K922 |
| Peptic ulcer disease | K27 |
| Upper GI bleed | K250, K252, K254, K256, K260, K262, K264, K266 |
| Lower/ unspecified GI bleed | K270, K272, K274, K276, K280, K282, K284, K286, K5521, K566, K5701, K5711, K5713, K5721, K5731, K5733, K5741, K5751, K5753, K5781, K5791, K5793, K625, K631, K661, K920, K921, K922 |
| Urogenital bleed | R319, R319, R310, R311, R312 |
| Other bleeds | I312, M250, R58, R042, R049, R0481, R0489 |
| History of cancer | C00-C97 |
| **Concomitant medications** | **ATC codes** |
| Meglitinides | A10BX02, A10BX03, A10BX05, A10BX08 |
| Alpha glucosidase inhibitor | A10BF |
| Thiazide | C03AA |
| Beta-blockers | C07A |
| Calcium channel blockers | C08 |
| All antidiabetic medications except insulin | A10B |
| Insulin | A10A |
| Low intensity statins | C10AA (depends on type and dosage of statin) |
| High intensity statins | C10AA (depends on type and dosage of statin) |
| CNS stimulants | A08AA, N06BA |
| Estrogens, progestins, androgens | G03C, G03D, G03B |
| Angiogenesis inhibitors | L01EX01, L01EX02, L01EX03, L01EX04, L01EX05, L01EX07, L01EX08, L01EK01, L01FG01, L01FG02 |
| Oral immunosuppressants | L04AX01, L04AX03, L04AX05, L04AA06, L04AA10, L04AA13, L04AA37, L04AA27, L04AA29, L04AD02, M01CC02, L04AD01, L04AD01, A07EC01, P01BA02, J01AA08 |
| Other direct thrombin inhibitors (lepirudin, desirudin, argatroban) | B01AE01, B01AE02, B01AE03 |
| Ticagrelor | B01AC24 |
| Antibiotics | J01 |
| Aspirin | B01AC06 |
| Other antiplatelet agents | B01AC |
| P-gp inhibitors | C01BD01, J01FA10, N03AX24, C07AG02, J01FA09, V03AX03, L04AD01, J05AP07, C05CA03, C01BD07, A16AX10, J01FA01, J05AP57, J02AC02, J02AB02, L01EH01, J05AP51-3, L01EB04, C01BC03, C01BA01, C01EB18, J05AE03, L02BA01, B01AC24, L01EC01, C08DA01 |
| Other gastroprotective agents | A02BX |
| Proton pump inhibitors | A02BC |
| H2 receptor antagonist | A02BA |
| Vitamin K therapy | B02BA01 |
| Alpha-blockers | G04CA01 |
| Prasugrel | B01AC22 |
| Antihypertensive drugs | C09 |
| **Procedure or tests** |  |
| Cerebrovascular procedure | M6630, M6631, M6633, M6636, M1661, M1662, M1663, M1664, M1665, M1666, M1667, M1668, M1669, M1673, M1674, M1675, M6593, M6599, M6601 |
| Dialysis | O7020, O7061, O7062 |
| CABG or PTCA or stent | O1641, OA641, O1640, OA640, O1642, OA642, O1648, OA648, O1649, OA649, O1647, OA647, M6551, M6553, M6554, M6552, M6561, M6563, M6562, M6564, M6565, M6566, M6567 |
| Cardiovascular stress test | E6542, E6543 |
| Echocardiogram | EA431, EA432, EA433, EA434, E9431, E9432, E9433, E9434, E9435, E9436, E9437, E9438, E9439, EB430, EB431, EB432, EB433, EB434, EB435, EB436, EB610, EB611, EB612 |
| BNP tests | CZ208 |
| Cardiac biomarkers tests (troponin, CK-MBs, Myoglobin, CPK) | D4021, D4022, D4023, D4040, D4061, D4062, D4011, D4012, D4013, B2630 |
| Ambulatory blood pressure monitoring tests | E6547, E6548 |
| Colonoscopy | E7660, E7670, E7680 |
| Fecal occult blood (FOB) test | D0318, D0319, D0320 |
| Mammogram | G2701, G2702, G2703, G2704, G2705, G2706, G2707, G2708, G2709 |
| PSA test or Prostate exam for DRE | D4300 |
| D-dimer tests | D1071-D1073 |
| CRP, high-sensitivity CRP tests | D0111, D0112, D0113, D0114 |
| PT or aPTT tests | D1003, D1004 |
| Bleeding time tests | D1001 |
| Lipid tests ordered | D2261, D2262, D2263, D2264, D2265, D2266 |
| INR (prothrombin) tests ordered | D1008 |
| **Measures of health status** |  |
| HAS-BLED score | Calculated according to Pisters et al., 2010 |
| CHA_2_DS_2_-VASc score | Calculated according to Olesen et al., 2012 |
| CCI score | Calculated according to Charlson et al., 2022 |

**Abbreviations:** ATC, Anatomical Therapeutic Chemical Classification; ICD-10, International Classification of Diseases 10^th^ revision; HIRA, Health Insurance Review and Assessment Service of South Korea; VTE, Venous thromboembolism; GI, Gastrointestinal; CNS, Central nervous system; P-gp, P-glycoprotein; CABG, Coronary artery bypass grafting; PTCA, Percutaneous transluminal coronary angioplasty; BNP, Brain natriuretic peptide; CK-MB, Creatine kinase MB isoenzyme; CPK, Creatine phosphokinase; PSA, Prostate-specific antigen; DRE, Digital rectal examination; CRP, C-reactive protein; PT, Prothrombin time; aPTT, Activated partial thromboplastin time; INR, International normalized ratio.

Table S3. Codes used to identify the safety outcome: Major bleeding

| **Outcome** | **Setting** | **Description** | **ICD-10 code or procedure code** |
| --- | --- | --- | --- |
| Intracranial bleeding | Primary diagnosis + Hospitalization | Subarachnoid and intracranial hemorrhage | I60-I62 |
|  | Procedure | Brain CT | HA441, HA451, HA461, HA471, HA851 |
|  |  | Brain MRI | HE101, HE102, HE135, HE201, HE202, HE235, HE301, HE302, HE401, HE402, HE501, HE502, HE535 |
| GI bleeding | Primary diagnosis + Hospitalization | Lower + Upper gastrointestinal bleeding | K250, K252, K254, K256, K260, K262, K264, K266, K270, K272, K274, K276, K280, K282, K284, K286, K5521, K566, K5701, K5711, K5713, K5721, K5731, K5733, K5741, K5751, K5753, K5781, K5791, K5793, K625, K631, K661, K920, K921, K922 |
| Other bleeding | Primary diagnosis + Hospitalization | Hemopericardium and others | I312, M250, R58, R042, R049, R0481, R0489 |

**Abbreviations:** ICD-10, International Classification of Diseases 10^th^ revision

Table S4. Constructing the emulation cohort with the same criteria of the AMPLIFY trial

| **Step** | **Definitions** | **No. of Patients Excluded** | **No. of Patients Remained** |
| --- | --- | --- | --- |
| **Inclusion** | | | |
|  | Adult patients who newly initiated apixaban or warfarin with a diagnosis of VTE between May 2015 and August 2020 | - | 15,577 |
| **Exclusion** | | | |
| 1 | Women of childbearing potential, women who are pregnant or breastfeeding | 106 | 15,471 |
| 2 | Thrombectomy, insertion of a caval filter, or use of a fibrinolytic agent to treat current episode of VTE | 3,677 | 11,794 |
| 3 | Mechanical valve or atrial fibrillation or atrial flutter | 438 | 11,356 |
| 4 | Conditions for which serious bleeding may occur | 1,221 | 10,135 |
| 5 | Active and clinically significant liver disease | 1,169 | 8,966 |
| 6 | Bacterial endocarditis | 1 | 8,965 |
| 7 | Uncontrolled hypertension | 34 | 8,931 |
| 8 | Platelet count < 100,000/mm^3^ | 23 | 8,908 |
| 9 | Hemoglobin < 9g/dL | 249 | 8,659 |
| 10 | Serum creatinine > 2.5mg/dL or calculated creatinine clearance < 25ml/min | 333 | 8,326 |
| 11 | Subject requiring aspirin > 165mg/day at randomization | 8 | 8,318 |
| 12 | Subject requiring dual antiplatelet therapy at randomization | 735 | 7,583 |
| 13 | Life expectancy < 6 months (defined as CCI > 10) | 68 | 7,515 |
| 14 | Active bleeding or high risk for bleeding contraindicating treatment with LMWH and VKA | 39 | 7,476 |
|  |  | **Unmatched cohort** | **Matched cohort** |
|  | New users of **apixaban** (treatment) | 3,937 | 1,753 |
|  | New users of **warfarin** (comparator) | 3,539 | 1,753 |

**Abbreviations:** VTE, Venous thromboembolism; LMWH, Low-molecular weight heparin; VKA, Vitamin-K antagonist

Table S5. Constructing the emulation cohort with the same criteria of the RE-COVER II trial

| **Step** | **Definitions** | **No. of Patients Excluded** | **No. of Patients Remained** |
| --- | --- | --- | --- |
| **Inclusion** | | | |
|  | Adult patients who newly initiated dabigatran or warfarin with a diagnosis of VTE between May 2015 and August 2020 | - | 10,447 |
| **Exclusion** | | | |
| 1 | PE with embolectomy, thrombolytic therapy, suspected source of PE is other than blood clots from the legs | 1,475 | 8,972 |
| 2 | Actual or anticipated use of vena cava filter | 962 | 8,010 |
| 3 | Patients who in the investigator’s judgement are perceived as having an excessive risk of bleeding | 465 | 7,545 |
| 4 | Known anemia | 197 | 7,348 |
| 5 | Need of anticoagulant treatment for disorders other than VTE | 1,437 | 5,911 |
| 6 | Recent unstable cardiovascular disease | 221 | 5,690 |
| 7 | Liver disease expected to have any potential impact on survival (severe liver conditions) | 651 | 5,039 |
| 8 | Severe renal impairment (CrCL <30 mL per minute) | 205 | 4,834 |
| 9 | Contraindications to anticoagulant therapy:  Malignant or severe, uncontrolled hypertension, Active infective endocarditis, Mechanical prosthetic heart valve | 22 | 4,812 |
| 10 | Women who are pregnant, nursing, or of childbearing potential | 60 | 4,752 |
| 11 | Patients considered unsuitable for inclusion by the investigator (drug abuse or dependence) | 63 | 4,689 |
| 12 | Patients considered unsuitable for inclusion by the investigator (patients with life expectancy < 6 months) | 6 | 4,683 |
|  |  | **Unmatched cohort** | **Matched cohort** |
|  | New users of **dabigatran** (treatment) | 1,475 | 1,226 |
|  | New users of **warfarin** (comparator) | 3,208 | 1,226 |

**Abbreviations:** VTE, Venous thromboembolism; PE, Pulmonary embolism; CrCL, Creatinine clearance

Table S6. Constructing the emulation cohort with the same criteria of the Hokusai-VTE trial

| **Step** | **Definitions** | **No. of Patients Excluded** | **No. of Patients Remained** |
| --- | --- | --- | --- |
| **Inclusion** | | | |
|  | Adult patients who newly initiated edoxaban or warfarin with a diagnosis of VTE between February 2016 and August 2020 | - | 9,910 |
| **Exclusion** | | | |
| 1 | Thrombectomy, insertion of a caval filter, or use of a fibrinolytic agent to treat the current episode of DVT and/or PE | 2,088 | 7,822 |
| 2 | Indication for warfarin other than DVT and/or PE | 1,553 | 6,269 |
| 3 | Calculated CrCL < 30 mL/min | 259 | 6,010 |
| 4 | Significant liver disease | 727 | 5,283 |
| 5 | Active bleeding or high risk for bleeding contraindicating treatment with (LMW) heparin or warfarin | 304 | 4,979 |
| 6 | Uncontrolled hypertension as judged by the investigator | 23 | 4,956 |
| 7 | Women of childbearing potential without proper contraceptive measures, and women who are pregnant or breast feeding | 45 | 4,911 |
| 8 | Treatment with the potent P-gp inhibitors ritonavir, nelfinavir, indinavir, or saquinavir anticipated to continue during the study | 0 | 4,911 |
| 9 | Systemic use of the strong P-gp inhibitors ketoconazole, itraconazole, erythromycin, azithromycin or clarithromycin at the time of randomization | 269 | 4,642 |
| 10 | Life expectancy < 3 months (defined as CCI > 10) | 34 | 4,608 |
|  |  | **Unmatched cohort** | **Matched cohort** |
|  | New users of **edoxaban** (treatment) | 2,063 | 1,505 |
|  | New users of **warfarin** (comparator) | 2,545 | 1,505 |

**Abbreviations:** VTE, Venous thromboembolism; DVT, Deep vein thrombosis; PE, Pulmonary embolism; CrCL, Creatinine clearance; P-gp, P-glycoprotein

Table S7. Constructing the emulation cohort with the same criteria of the EINSTEIN-DVT trial

| **Step** | **Definitions** | **No. of Patients Excluded** | **No. of Patients Remained** |
| --- | --- | --- | --- |
| **Inclusion** | | | |
|  | Adult patients who newly initiated rivaroxaban or warfarin with a diagnosis of VTE between January 2013 and August 2020 | - | 19,553 |
| **Exclusion** | | | |
| 1 | Thrombectomy, insertion of a caval filter, or use of a fibrinolytic agent to treat the current episode of DVT or PE | 6,228 | 13,325 |
| 2 | Other indications for VKA than DVT and/or PE | 1,638 | 11,687 |
| 3 | Creatinine clearance <30 ml/min | 301 | 11,386 |
| 4 | Significant liver disease (e.g. acute hepatitis, chronic active hepatitis, cirrhosis) or ALAT>3x ULN | 1,352 | 10,034 |
| 5 | Bacterial endocarditis | 2 | 10,032 |
| 6 | Active bleeding or high risk for bleeding contraindicating treatment with enoxaparin or VKA | 441 | 9,591 |
| 7 | Systolic blood pressure >180 mgHg or diastolic blood pressure >110mgHg | 50 | 9,541 |
| 8 | Childbearing potential without proper contraceptive measures, pregnancy, or breastfeeding | 63 | 9,478 |
| 9 | Concomitant use of strong CYP3A4 inhibitors (e.g. HIV protease inhibitors, systemic ketoconazole) or strong CYP3A4 inducers rifampin | 346 | 9,132 |
| 10 | Life expectancy <3 months (defined as CCI > 10) | 64 | 9,068 |
|  |  | **Unmatched cohort** | **Matched cohort** |
|  | New users of **rivaroxaban** (treatment) | 6,368 | 2,135 |
|  | New users of **warfarin** (comparator) | 2,700 | 2,135 |

**Abbreviations:** PE, Pulmonary embolism; DVT, Deep vein thrombosis; VKA, Vitamin-K antagonist; ALAT, Alanine transaminase; ULN, Upper limit of normal; CrCL, Creatinine clearance; CYP, Cytochrome P450; HIV, Human immunodeficiency virus; CCI, Charlson comorbidity index.

Table S8. Constructing the emulation cohort with the same criteria of the EINSTEIN-PE trial

| **Step** | **Definitions** | **No. of Patients Excluded** | **No. of Patients Remained** |
| --- | --- | --- | --- |
| **Inclusion** | | | |
|  | Adult patients who newly initiated rivaroxaban or warfarin with a diagnosis of PE between January 2013 and August 2020 | - | 27,156 |
| **Exclusion** | | | |
| 1 | Thrombectomy, insertion of a caval filter, or use of a fibrinolytic agent to treat the current episode of DVT or PE | 4,070 | 23,086 |
| 2 | Other indications for VKA than DVT and/or PE | 4,709 | 18,377 |
| 3 | Creatinine clearance <30 ml/min | 311 | 18,066 |
| 4 | Significant liver disease (e.g. acute hepatitis, chronic active hepatitis, cirrhosis) or ALAT>3x ULN | 2,009 | 16,057 |
| 5 | Bacterial endocarditis | 12 | 16,045 |
| 6 | Active bleeding or high risk for bleeding contraindicating treatment with enoxaparin or VKA | 1,010 | 15,035 |
| 7 | Systolic blood pressure >180 mgHg or diastolic blood pressure >110mgHg | 61 | 14,974 |
| 8 | Childbearing potential without proper contraceptive measures, pregnancy, or breastfeeding | 131 | 14,843 |
| 9 | Concomitant use of strong CYP3A4 inhibitors (e.g. HIV protease inhibitors, systemic ketoconazole) or strong CYP3A4 inducers rifampin | 1,096 | 13,747 |
| 10 | Life expectancy <3 months (defined as CCI > 10) | 140 | 13,607 |
|  |  | **Unmatched cohort** | **Matched cohort** |
|  | New users of **rivaroxaban** (treatment) | 9,376 | 2,801 |
|  | New users of **warfarin** (comparator) | 4,191 | 2,801 |

**Abbreviations:** PE, Pulmonary embolism; DVT, Deep vein thrombosis; VKA, Vitamin-K antagonist; ALAT, Alanine transaminase; ULN, Upper limit of normal; CrCL, Creatinine clearance; CYP, Cytochrome P450; HIV, Human immunodeficiency virus; CCI, Charlson comorbidity index.

Table S9. Baseline characteristics of AMPLIFY emulation cohort

| **Variables** | **Unmatched cohort** | |  | **PS Matched cohort** | |  |
| --- | --- | --- | --- | --- | --- | --- |
|  | **Apixaban**  **(N=3,937)** | **Warfarin**  **(N=3,539)** | **aSD** | **Apixaban**  **(N=1,753)** | **Warfarin**  **(N=1,753)** | **aSD** |
| **Median days of follow-up (IQR)** |  |  |  | 109 (35-241) | 111 (25-344) |  |
| **Age, mean (SD), y** | 71.9 (15.6) | 67.2 (17) | 0.29 | 68.9 (17) | 68.9 (16.4) | 0.01 |
| **Male sex, n (%)** | 1434 (36.4) | 1561 (44.1) | 0.16 | 744 (42.4) | 715 (40.8) | 0.03 |
| **Health status relevant with the use of anticoagulant, mean (SD)** |  |  |  |  |  |  |
| CHA_2_DS_2_ VASc score | 2.8 (1.6) | 2.5 (1.7) | 0.21 | 2.6 (1.6) | 2.6 (1.6) | 0.01 |
| HAS-BLED Score | 2.3 (1) | 2.2 (1.1) | 0.13 | 2.2 (1.1) | 2.2 (1.1) | 0.02 |
| **Number of laboratory tests, mean (SD)** |  |  |  |  |  |  |
| Number of brain natriuretic peptide tests | 0.1 (0.4) | 0.2 (0.5) | 0.20 | 0.3 (0.5) | 0.2 (0.6) | 0.02 |
| Number of cardiac biomarkers tests | 1.3 (1.1) | 1.2 (1.1) | 0.16 | 1.2 (1) | 1.3 (1.2) | 0.02 |
| Number of ambulatory blood pressure monitoring tests | 0.4 (0.6) | 0.3 (0.6) | 0.14 | 0.3 (0.6) | 0.3 (0.6) | 0.01 |
| Number of D-dimer tests | 0.9 (0.8) | 0.3 (0.8) | 0.69 | 0.6 (0.7) | 0.6 (1) | 0.01 |
| Number of C-reactive protein, high-sensitivity CRP tests | 1.8 (2.2) | 0.7 (1.6) | 0.61 | 1.2 (1.9) | 1.2 (2) | 0.01 |
| Number of prothrombin time or aPTT tests | 1.4 (1.5) | 0.5 (1.2) | 0.61 | 1 (1.4) | 0.9 (1.5) | 0.02 |
| Number of Bleeding time tests | 0 (0.2) | 0 (0.2) | 0.10 | 0 (0.2) | 0 (0.2) | 0.02 |
| Number of INR tests | 0 (0) | 0 (0.1) | 0.08 | 0 (0) | 0 (0) | 0.03 |
| Number of lipid tests | 0.9 (1.3) | 0.3 (0.9) | 0.51 | 0.6 (1.2) | 0.6 (1.1) | 0.02 |
| **Comorbidity, n (%)** |  |  |  |  |  |  |
| Deep vein thrombosis only | 966 (24.5) | 1404 (39.7) | 0.33 | 594 (33.9) | 596 (34) | 0.00 |
| Pulmonary embolism with or without deep vein thrombosis | 2971 (75.5) | 2135 (60.3) | 0.33 | 1159 (66.1) | 1157 (66) | 0.00 |
| Previous venous thromboembolism | 564 (14.3) | 710 (20.1) | 0.15 | 316 (18) | 313 (17.9) | 0.00 |
| Active cancer | 536 (13.6) | 364 (10.3) | 0.10 | 213 (12.2) | 192 (11) | 0.04 |
| Hemorrhagic stroke or other cerebrovascular disease or cerebrovascular procedure | 412 (10.5) | 356 (10.1) | 0.01 | 187 (10.7) | 174 (9.9) | 0.02 |
| Major trauma potentially causing prolonged immobilization | 50 (1.3) | 31 (0.9) | 0.04 | 16 (0.9) | 20 (1.1) | 0.02 |
| Chronic kidney disease Stage 1-2 | 9 (0.2) | 7 (0.2) | 0.01 | 3 (0.2) | 3 (0.2) | 0.00 |
| Chronic kidney disease Stage 3-6 | 25 (0.6) | 35 (1) | 0.04 | 14 (0.8) | 16 (0.9) | 0.01 |
| Acute kidney injury | 82 (2.1) | 64 (1.8) | 0.02 | 26 (1.5) | 33 (1.9) | 0.03 |
| Bladder stones or kidney stones | 60 (1.5) | 55 (1.6) | 0.00 | 25 (1.4) | 24 (1.4) | 0.01 |
| Alcohol abuse/dependence or drug abuse/dependence | 34 (0.9) | 28 (0.8) | 0.01 | 17 (1) | 18 (1) | 0.01 |
| Atherosclerosis or cardiac conduction disorders or Other CVD | 788 (20) | 703 (19.9) | 0.00 | 335 (19.1) | 337 (19.2) | 0.00 |
| Diabetes with complication | 386 (9.8) | 288 (8.1) | 0.06 | 137 (7.8) | 148 (8.4) | 0.02 |
| Systemic embolism | 4 (0.1) | 4 (0.1) | 0.00 | 2 (0.1) | 2 (0.1) | 0.00 |
| Coagulation defects | 24 (0.6) | 19 (0.5) | 0.01 | 10 (0.6) | 10 (0.6) | 0.00 |
| Diabetes | 667 (16.9) | 529 (14.9) | 0.05 | 256 (14.6) | 277 (15.8) | 0.03 |
| Intracranial or retroperitoneal hemorrhage | 34 (0.9) | 20 (0.6) | 0.04 | 18 (1) | 11 (0.6) | 0.04 |
| Peptic ulcer disease | 106 (2.7) | 123 (3.5) | 0.05 | 59 (3.4) | 59 (3.4) | 0.00 |
| Upper gastrointestinal bleed | 7 (0.2) | 7 (0.2) | 0.01 | 3 (0.2) | 3 (0.2) | 0.00 |
| Lower/ unspecified gastrointestinal bleed | 33 (0.8) | 15 (0.4) | 0.05 | 11 (0.6) | 8 (0.5) | 0.02 |
| Urogenital bleed | 29 (0.7) | 29 (0.8) | 0.01 | 16 (0.9) | 13 (0.7) | 0.02 |
| Other bleeds | 29 (0.7) | 22 (0.6) | 0.01 | 11 (0.6) | 10 (0.6) | 0.01 |
| **Concomitant medication, n (%)** |  |  |  |  |  |  |
| Meglitinides | 8 (0.2) | 16 (0.5) | 0.04 | 5 (0.3) | 3 (0.2) | 0.02 |
| Alpha glucosidase inhibitors | 22 (0.6) | 25 (0.7) | 0.02 | 14 (0.8) | 16 (0.9) | 0.01 |
| Thiazide | 745 (18.9) | 658 (18.6) | 0.01 | 323 (18.4) | 322 (18.4) | 0.00 |
| Beta blockers | 1045 (26.5) | 887 (25.1) | 0.03 | 445 (25.4) | 455 (26) | 0.01 |
| Calcium channel blockers | 1855 (47.1) | 1480 (41.8) | 0.11 | 750 (42.8) | 756 (43.1) | 0.01 |
| All antidiabetic medications except Insulin | 766 (19.5) | 578 (16.3) | 0.08 | 290 (16.5) | 315 (18) | 0.04 |
| Insulin | 622 (15.8) | 481 (13.6) | 0.06 | 250 (14.3) | 253 (14.4) | 0.01 |
| Low Intensity Statins | 49 (1.2) | 42 (1.2) | 0.01 | 16 (0.9) | 14 (0.8) | 0.01 |
| High Intensity Statins | 148 (3.8) | 118 (3.3) | 0.02 | 55 (3.1) | 59 (3.4) | 0.01 |
| Central nervous system stimulants | 13 (0.3) | 9 (0.3) | 0.01 | 5 (0.3) | 5 (0.3) | 0.00 |
| Estrogens or progestins or androgens | 91 (2.3) | 92 (2.6) | 0.02 | 38 (2.2) | 44 (2.5) | 0.02 |
| Angiogenesis inhibitors | 32 (0.8) | 21 (0.6) | 0.03 | 13 (0.7) | 15 (0.9) | 0.01 |
| Oral Immunosuppressants | 134 (3.4) | 139 (3.9) | 0.03 | 64 (3.7) | 70 (4) | 0.02 |
| Other direct thrombin inhibitors | 2 (0.1) | 4 (0.1) | 0.02 | 1 (0.1) | 0 (0) | 0.03 |
| Clopidogrel | 354 (9) | 287 (8.1) | 0.03 | 143 (8.2) | 141 (8) | 0.00 |
| Antibiotics | 3387 (86) | 3033 (85.7) | 0.01 | 1493 (85.2) | 1500 (85.6) | 0.01 |
| Aspirin | 863 (21.9) | 928 (26.2) | 0.10 | 423 (24.1) | 426 (24.3) | 0.00 |
| Other antiplatelet agents | 1293 (32.8) | 1078 (30.5) | 0.05 | 520 (29.7) | 556 (31.7) | 0.05 |
| P-glycoprotein inhibitors | 936 (23.8) | 825 (23.3) | 0.01 | 426 (24.3) | 434 (24.8) | 0.01 |
| Other gastroprotective agents | 1784 (45.3) | 1626 (45.9) | 0.01 | 775 (44.2) | 773 (44.1) | 0.00 |
| Proton pump inhibitor | 2456 (62.4) | 1900 (53.7) | 0.18 | 1010 (57.6) | 1006 (57.4) | 0.01 |
| H_2_ receptor antagonist | 2849 (72.4) | 2691 (76) | 0.08 | 1310 (74.7) | 1325 (75.6) | 0.02 |
| Vitamin K therapy | 72 (1.8) | 203 (5.7) | 0.21 | 51 (2.9) | 59 (3.4) | 0.03 |
| Prasugrel | 1 (0) | 3 (0.1) | 0.03 | 0 (0) | 1 (0.1) | 0.03 |
| Alpha blockers | 789 (20) | 618 (17.5) | 0.07 | 336 (19.2) | 314 (17.9) | 0.03 |
| Antihypertensives | 1844 (46.8) | 1499 (42.4) | 0.09 | 771 (44) | 768 (43.8) | 0.00 |
| Number of days on antihypertensives during baseline | 97.6 (85.5) | 87.6 (86.1) | 0.12 | 89.5 (86) | 90.8 (86) | 0.02 |
| **Procedure or tests, n (%)** |  |  |  |  |  |  |
| Previous cardiac procedure | 2 (0.1) | 4 (0.1) | 0.02 | 1 (0.1) | 1 (0.1) | 0.00 |
| Cardiovascular stress test | 25 (0.6) | 29 (0.8) | 0.02 | 13 (0.7) | 18 (1) | 0.03 |
| Echocardiogram | 1345 (34.2) | 717 (20.3) | 0.32 | 480 (27.4) | 481 (27.4) | 0.00 |
| Colonoscopy | 349 (8.9) | 295 (8.3) | 0.02 | 150 (8.6) | 152 (8.7) | 0.00 |
| Fecal occult blood (FOB) test | 522 (13.3) | 186 (5.3) | 0.28 | 140 (8) | 156 (8.9) | 0.03 |
| Mammogram | 70 (1.8) | 66 (1.9) | 0.01 | 42 (2.4) | 34 (1.9) | 0.03 |
| Prostate-specific antigen test or Prostate exam for DRE | 300 (7.6) | 111 (3.1) | 0.20 | 91 (5.2) | 95 (5.4) | 0.01 |

**Abbreviations:** aPTT, Activated partial thromboplastin time; INR, International normalized ratio; DRE, Digital rectal examination.

Table S10. Baseline characteristics of RE-COVER II emulation cohort

| **Variables** | **Unmatched cohort** | |  | **PS Matched cohort** | |  |
| --- | --- | --- | --- | --- | --- | --- |
|  | **Dabigatran**  **(N=1,475)** | **Warfarin**  **(N=3,208)** | **aSD** | **Dabigatran**  **(N=1,226)** | **Warfarin**  **(N=1,226)** | **aSD** |
| **Median days of follow-up (IQR)** |  |  |  | 100 (26-187) | 53 (13-206) |  |
| **Age, mean (SD), y** | 68.8 (15.9) | 65.7 (17.5) | 0.18 | 68 (16.3) | 68 (16.3) | 0.00 |
| **Sex, n (%)** | 582 (39.5) | 1422 (44.3) | 0.10 | 503 (41) | 501 (40.9) | 0.00 |
| **Health status relevant with the use of anticoagulant, mean (SD)** |  |  |  |  |  |  |
| CHA_2_DS_2_ VASc score | 2.4 (1.5) | 2.2 (1.6) | 0.12 | 2.3 (1.5) | 2.3 (1.5) | 0.00 |
| HAS-BLED Score | 2.2 (1) | 2.1 (1) | 0.11 | 2.1 (1) | 2.1 (1) | 0.00 |
| **Number of laboratory tests, mean (SD)** |  |  |  |  |  |  |
| Number of brain natriuretic peptide tests | 0.3 (0.5) | 0.2 (0.5) | 0.11 | 0.3 (0.5) | 0.2 (0.5) | 0.01 |
| Number of cardiac biomarkers tests | 1.2 (1) | 1.1 (1) | 0.15 | 1.2 (1) | 1.2 (0.9) | 0.02 |
| Number of ambulatory blood pressure monitoring tests | 0.3 (0.6) | 0.3 (0.5) | 0.11 | 0.3 (0.6) | 0.3 (0.5) | 0.00 |
| Number of D-dimer tests | 0.4 (0.7) | 0.3 (0.7) | 0.11 | 0.4 (0.7) | 0.4 (0.7) | 0.02 |
| Number of C-reactive protein, high-sensitivity CRP tests | 0.8 (1.5) | 0.7 (1.4) | 0.09 | 0.7 (1.5) | 0.7 (1.4) | 0.00 |
| Number of prothrombin time or aPTT tests | 0.6 (1.1) | 0.5 (1.1) | 0.06 | 0.6 (1.1) | 0.6 (1) | 0.02 |
| Number of Bleeding time tests | 0 (0.2) | 0 (0.2) | 0.02 | 0 (0.2) | 0 (0.2) | 0.01 |
| Number of INR tests | 0 (0) | 0 (0.1) | 0.10 | 0 (0) | 0 (0) | N/A |
| Number of lipid tests | 0.4 (0.8) | 0.3 (0.8) | 0.08 | 0.4 (0.8) | 0.4 (0.8) | 0.01 |
| **Comorbidity, n (%)** |  |  |  |  |  |  |
| Deep vein thrombosis only | 373 (25.3) | 1601 (49.9) | 0.53 | 362 (29.5) | 375 (30.6) | 0.02 |
| Pulmonary embolism with or without deep vein thrombosis | 1102 (74.7) | 1607 (50.1) | 0.53 | 864 (70.5) | 851 (69.4) | 0.02 |
| Previous venous thromboembolism | 263 (17.8) | 683 (21.3) | 0.09 | 231 (18.8) | 223 (18.2) | 0.02 |
| Active cancer | 58 (3.9) | 157 (4.9) | 0.05 | 52 (4.2) | 54 (4.4) | 0.01 |
| Hemorrhagic stroke or other cerebrovascular disease or cerebrovascular procedure | 101 (6.8) | 169 (5.3) | 0.07 | 72 (5.9) | 73 (6) | 0.00 |
| Major trauma potentially causing prolonged immobilization | 26 (1.8) | 38 (1.2) | 0.05 | 18 (1.5) | 16 (1.3) | 0.01 |
| Chronic kidney disease Stage 1-2 | 3 (0.2) | 3 (0.1) | 0.03 | 1 (0.1) | 2 (0.2) | 0.02 |
| Chronic kidney disease Stage 3-6 | 7 (0.5) | 19 (0.6) | 0.02 | 4 (0.3) | 4 (0.3) | 0.00 |
| Acute kidney injury | 11 (0.7) | 58 (1.8) | 0.10 | 11 (0.9) | 12 (1) | 0.01 |
| Bladder stones or kidney stones | 18 (1.2) | 44 (1.4) | 0.01 | 13 (1.1) | 17 (1.4) | 0.03 |
| Alcohol abuse/dependence or drug abuse/dependence | 0 (0.0) | 0 (0.0) | N/A | 0 (0.0) | 0 (0.0) | N/A |
| Atherosclerosis or cardiac conduction disorders or Other CVD | 183 (12.4) | 463 (14.4) | 0.06 | 154 (12.6) | 156 (12.7) | 0.01 |
| Diabetes with complication | 121 (8.2) | 247 (7.7) | 0.02 | 94 (7.7) | 98 (8) | 0.01 |
| Systemic embolism | 1 (0.1) | 9 (0.3) | 0.05 | 1 (0.1) | 0 (0) | 0.04 |
| Coagulation defects | 9 (0.6) | 42 (1.3) | 0.07 | 8 (0.7) | 12 (1) | 0.04 |
| Diabetes | 210 (14.2) | 469 (14.6) | 0.01 | 170 (13.9) | 163 (13.3) | 0.02 |
| Intracranial or retroperitoneal hemorrhage | 0 (0.0) | 0 (0.0) | N/A | 0 (0.0) | 0 (0.0) | N/A |
| Peptic ulcer disease | 50 (3.4) | 101 (3.1) | 0.01 | 42 (3.4) | 46 (3.8) | 0.02 |
| Upper gastrointestinal bleed | 0 (0.0) | 0 (0.0) | N/A | 0 (0.0) | 0 (0.0) | N/A |
| Lower/ unspecified gastrointestinal bleed | 0 (0.0) | 0 (0.0) | N/A | 0 (0.0) | 0 (0.0) | N/A |
| Urogenital bleed | 0 (0.0) | 0 (0.0) | N/A | 0 (0.0) | 0 (0.0) | N/A |
| Other bleeds | 0 (0.0) | 0 (0.0) | N/A | 0 (0.0) | 0 (0.0) | N/A |
| **Concomitant medication, n (%)** |  |  |  |  |  |  |
| Meglitinides | 2 (0.1) | 16 (0.5) | 0.07 | 2 (0.2) | 2 (0.2) | 0.00 |
| Alpha glucosidase inhibitors | 9 (0.6) | 21 (0.7) | 0.01 | 7 (0.6) | 7 (0.6) | 0.00 |
| Thiazide | 262 (17.8) | 549 (17.1) | 0.02 | 206 (16.8) | 213 (17.4) | 0.02 |
| Beta blockers | 382 (25.9) | 759 (23.7) | 0.05 | 316 (25.8) | 297 (24.2) | 0.04 |
| Calcium channel blockers | 647 (43.9) | 1301 (40.6) | 0.07 | 511 (41.7) | 516 (42.1) | 0.01 |
| All antidiabetic medications except Insulin | 242 (16.4) | 517 (16.1) | 0.01 | 192 (15.7) | 199 (16.2) | 0.02 |
| Insulin | 176 (11.9) | 419 (13.1) | 0.03 | 147 (12) | 138 (11.3) | 0.02 |
| Low Intensity Statins | 15 (1) | 35 (1.1) | 0.01 | 11 (0.9) | 13 (1.1) | 0.02 |
| High Intensity Statins | 71 (4.8) | 126 (3.9) | 0.04 | 63 (5.1) | 45 (3.7) | 0.07 |
| Central nervous system stimulants | 6 (0.4) | 8 (0.2) | 0.03 | 2 (0.2) | 2 (0.2) | 0.00 |
| Estrogens or progestins or androgens | 44 (3) | 77 (2.4) | 0.04 | 31 (2.5) | 32 (2.6) | 0.01 |
| Angiogenesis inhibitors | 2 (0.1) | 8 (0.2) | 0.03 | 0 (0.0) | 0 (0.0) | N/A |
| Oral Immunosuppressants | 46 (3.1) | 129 (4) | 0.05 | 38 (3.1) | 38 (3.1) | 0.00 |
| Other direct thrombin inhibitors | 0 (0.0) | 0 (0.0) | N/A | 0 (0.0) | 0 (0.0) | N/A |
| Clopidogrel | 158 (10.7) | 379 (11.8) | 0.04 | 132 (10.8) | 128 (10.4) | 0.01 |
| Antibiotics | 1264 (85.7) | 2732 (85.2) | 0.02 | 1050 (85.6) | 1052 (85.8) | 0.01 |
| Aspirin | 352 (23.9) | 942 (29.4) | 0.13 | 310 (25.3) | 295 (24.1) | 0.03 |
| Other antiplatelet agents | 485 (32.9) | 1037 (32.3) | 0.01 | 401 (32.7) | 395 (32.2) | 0.01 |
| P-glycoprotein inhibitors | 364 (24.7) | 717 (22.4) | 0.06 | 301 (24.6) | 310 (25.3) | 0.02 |
| Other gastroprotective agents | 716 (48.5) | 1417 (44.2) | 0.09 | 579 (47.2) | 581 (47.4) | 0.00 |
| Proton pump inhibitor | 866 (58.7) | 1642 (51.2) | 0.15 | 688 (56.1) | 660 (53.8) | 0.05 |
| H_2_ receptor antagonist | 1118 (75.8) | 2420 (75.4) | 0.01 | 932 (76) | 913 (74.5) | 0.04 |
| Vitamin K therapy | 22 (1.5) | 167 (5.2) | 0.21 | 21 (1.7) | 19 (1.5) | 0.01 |
| Prasugrel | 2 (0.1) | 4 (0.1) | 0.00 | 1 (0.1) | 1 (0.1) | 0.00 |
| Alpha blockers | 236 (16) | 503 (15.7) | 0.01 | 187 (15.3) | 192 (15.7) | 0.01 |
| Antihypertensives | 560 (38) | 1234 (38.5) | 0.01 | 469 (38.3) | 469 (38.3) | 0.00 |
| Number of days on antihypertensives during baseline | 90.2 (87.2) | 79.7 (86.1) | 0.12 | 86.6 (87) | 85 (86.5) | 0.02 |
| **Procedure or tests, n (%)** |  |  |  |  |  |  |
| Previous cardiac procedure | 4 (0.3) | 14 (0.4) | 0.03 | 3 (0.2) | 2 (0.2) | 0.02 |
| Cardiovascular stress test | 11 (0.7) | 26 (0.8) | 0.01 | 9 (0.7) | 7 (0.6) | 0.02 |
| Echocardiogram | 256 (17.4) | 549 (17.1) | 0.01 | 215 (17.5) | 215 (17.5) | 0.00 |
| Colonoscopy | 105 (7.1) | 229 (7.1) | 0.00 | 85 (6.9) | 85 (6.9) | 0.00 |
| Fecal occult blood (FOB) test | 90 (6.1) | 149 (4.6) | 0.07 | 72 (5.9) | 62 (5.1) | 0.04 |
| Mammogram | 24 (1.6) | 49 (1.5) | 0.01 | 17 (1.4) | 15 (1.2) | 0.01 |
| Prostate-specific antigen test or Prostate exam for DRE | 65 (4.4) | 100 (3.1) | 0.07 | 51 (4.2) | 49 (4) | 0.01 |

**Abbreviations:** aPTT, Activated partial thromboplastin time; INR, International normalized ratio; DRE, Digital rectal examination.

Table S11. Baseline characteristics of Hokusai-VTE emulation cohort

| **Variables** | **Unmatched cohort** | |  | **PS Matched cohort** | |  |
| --- | --- | --- | --- | --- | --- | --- |
|  | **Edoxaban**  **(N=2,063)** | **Warfarin**  **(N=2,545)** | **aSD** | **Edoxaban**  **(N=1,505)** | **Warfarin**  **(N=1,505)** | **aSD** |
| **Median days of follow-up, (IQR)** |  |  |  | 86 (24-189) | 54 (13-210) |  |
| **Age, mean (SD), y** | 70.4 (15.2) | 67 (16.9) | 0.21 | 68.8 (16.1) | 68.8 (16.1) | 0.00 |
| **Sex, n (%)** | 770 (37.3) | 1103 (43.3) | 0.12 | 601 (39.9) | 595 (39.5) | 0.01 |
| **Health status relevant with the use of anticoagulant, mean (SD)** |  |  |  |  |  |  |
| CHA_2_DS_2_ VASc score | 2.5 (1.5) | 2.3 (1.5) | 0.15 | 2.4 (1.5) | 2.4 (1.5) | 0.01 |
| HAS-BLED Score | 2.3 (1) | 2.1 (1) | 0.12 | 2.2 (1) | 2.2 (1) | 0.02 |
| **Number of laboratory tests, mean (SD)** |  |  |  |  |  |  |
| Number of brain natriuretic peptide tests | 0.1 (0.4) | 0.3 (0.6) | 0.25 | 0.2 (0.5) | 0.2 (0.5) | 0.01 |
| Number of cardiac biomarkers tests | 1.3 (1) | 1.1 (1) | 0.12 | 1.2 (1) | 1.2 (1) | 0.00 |
| Number of ambulatory blood pressure monitoring tests | 0.4 (0.6) | 0.3 (0.6) | 0.14 | 0.3 (0.6) | 0.3 (0.6) | 0.01 |
| Number of D-dimer tests | 0.9 (0.8) | 0.4 (0.8) | 0.58 | 0.8 (0.7) | 0.8 (0.8) | 0.01 |
| Number of C-reactive protein, high-sensitivity CRP tests | 1.9 (2.2) | 0.9 (1.7) | 0.51 | 1.6 (1.8) | 1.5 (2) | 0.01 |
| Number of prothrombin time or aPTT tests | 1.5 (1.6) | 0.7 (1.3) | 0.52 | 1.2 (1.4) | 1.2 (1.4) | 0.02 |
| Number of Bleeding time tests | 0 (0.2) | 0 (0.2) | 0.04 | 0 (0.2) | 0 (0.2) | 0.02 |
| Number of INR tests | 0 (0) | 0 (0.1) | 0.07 | 0 (0) | 0 (0) | 0.00 |
| Number of lipid tests | 0.9 (1.3) | 0.5 (1) | 0.41 | 0.8 (1.1) | 0.8 (1.2) | 0.02 |
| **Comorbidity, n (%)** |  |  |  |  |  |  |
| Deep vein thrombosis only | 588 (28.5) | 1074 (42.2) | 0.29 | 516 (34.3) | 518 (34.4) | 0.00 |
| Pulmonary embolism with or without deep vein thrombosis | 1475 (71.5) | 1471 (57.8) | 0.29 | 989 (65.7) | 987 (65.6) | 0.00 |
| Previous venous thromboembolism | 361 (17.5) | 558 (21.9) | 0.11 | 295 (19.6) | 276 (18.3) | 0.03 |
| Active cancer | 364 (17.6) | 246 (9.7) | 0.23 | 159 (10.6) | 156 (10.4) | 0.01 |
| Hemorrhagic stroke or other cerebrovascular disease or cerebrovascular procedure | 116 (5.6) | 139 (5.5) | 0.01 | 84 (5.6) | 88 (5.8) | 0.01 |
| Major trauma potentially causing prolonged immobilization | 23 (1.1) | 28 (1.1) | 0.00 | 20 (1.3) | 13 (0.9) | 0.05 |
| Chronic kidney disease Stage 1-2 | 5 (0.2) | 4 (0.2) | 0.02 | 2 (0.1) | 2 (0.1) | 0.00 |
| Chronic kidney disease Stage 3-6 | 10 (0.5) | 21 (0.8) | 0.04 | 10 (0.7) | 10 (0.7) | 0.00 |
| Acute kidney injury | 31 (1.5) | 48 (1.9) | 0.03 | 24 (1.6) | 17 (1.1) | 0.04 |
| Bladder stones or kidney stones | 22 (1.1) | 31 (1.2) | 0.01 | 15 (1) | 17 (1.1) | 0.01 |
| Alcohol abuse/dependence or drug abuse/dependence | 14 (0.7) | 22 (0.9) | 0.02 | 13 (0.9) | 9 (0.6) | 0.03 |
| Atherosclerosis or cardiac conduction disorders or Other CVD | 324 (15.7) | 428 (16.8) | 0.03 | 222 (14.8) | 211 (14) | 0.02 |
| Diabetes with complication | 177 (8.6) | 219 (8.6) | 0.00 | 133 (8.8) | 127 (8.4) | 0.01 |
| Systemic embolism | 3 (0.1) | 4 (0.2) | 0.00 | 2 (0.1) | 1 (0.1) | 0.02 |
| Coagulation defects | 13 (0.6) | 28 (1.1) | 0.05 | 12 (0.8) | 6 (0.4) | 0.05 |
| Diabetes | 312 (15.1) | 395 (15.5) | 0.01 | 215 (14.3) | 222 (14.8) | 0.01 |
| Intracranial or retroperitoneal hemorrhage | 0 (0) | 0 (0) | N/A | 0 (0) | 0 (0) | N/A |
| Peptic ulcer disease | 55 (2.7) | 95 (3.7) | 0.06 | 49 (3.3) | 51 (3.4) | 0.01 |
| Upper gastrointestinal bleed | 0 (0) | 0 (0) | N/A | 0 (0) | 0 (0) | N/A |
| Lower/ unspecified gastrointestinal bleed | 0 (0) | 0 (0) | N/A | 0 (0) | 0 (0) | N/A |
| Urogenital bleed | 0 (0) | 0 (0) | N/A | 0 (0) | 0 (0) | N/A |
| Other bleeds | 0 (0) | 0 (0) | N/A | 0 (0) | 0 (0) | N/A |
| **Concomitant medication, n (%)** |  |  |  |  |  |  |
| Meglitinides | 6 (0.3) | 18 (0.7) | 0.06 | 5 (0.3) | 5 (0.3) | 0.00 |
| Alpha glucosidase inhibitors | 9 (0.4) | 18 (0.7) | 0.04 | 8 (0.5) | 7 (0.5) | 0.01 |
| Thiazide | 349 (16.9) | 440 (17.3) | 0.01 | 267 (17.7) | 255 (16.9) | 0.02 |
| Beta blockers | 542 (26.3) | 674 (26.5) | 0.01 | 402 (26.7) | 378 (25.1) | 0.04 |
| Calcium channel blockers | 905 (43.9) | 1110 (43.6) | 0.01 | 644 (42.8) | 634 (42.1) | 0.01 |
| All antidiabetic medications except Insulin | 362 (17.5) | 443 (17.4) | 0.00 | 249 (16.5) | 252 (16.7) | 0.01 |
| Insulin | 272 (13.2) | 367 (14.4) | 0.04 | 196 (13) | 196 (13) | 0.00 |
| Low Intensity Statins | 13 (0.6) | 25 (1) | 0.04 | 10 (0.7) | 10 (0.7) | 0.00 |
| High Intensity Statins | 97 (4.7) | 111 (4.4) | 0.02 | 71 (4.7) | 68 (4.5) | 0.01 |
| Central nervous system stimulants | 6 (0.3) | 6 (0.2) | 0.01 | 4 (0.3) | 5 (0.3) | 0.01 |
| Estrogens or progestins or androgens | 61 (3) | 58 (2.3) | 0.04 | 42 (2.8) | 43 (2.9) | 0.00 |
| Angiogenesis inhibitors | 32 (1.6) | 16 (0.6) | 0.09 | 9 (0.6) | 14 (0.9) | 0.04 |
| Oral Immunosuppressants | 67 (3.2) | 104 (4.1) | 0.05 | 56 (3.7) | 60 (4) | 0.01 |
| Other direct thrombin inhibitors | 1 (0) | 0 (0) | 0.03 | 0 (0) | 0 (0) | N/A |
| Clopidogrel | 282 (13.7) | 345 (13.6) | 0.00 | 197 (13.1) | 187 (12.4) | 0.02 |
| Antibiotics | 1792 (86.9) | 2153 (84.6) | 0.07 | 1285 (85.4) | 1276 (84.8) | 0.02 |
| Aspirin | 499 (24.2) | 751 (29.5) | 0.12 | 396 (26.3) | 385 (25.6) | 0.02 |
| Other antiplatelet agents | 737 (35.7) | 846 (33.2) | 0.05 | 544 (36.1) | 531 (35.3) | 0.02 |
| P-glycoprotein inhibitors | 376 (18.2) | 504 (19.8) | 0.04 | 321 (21.3) | 307 (20.4) | 0.02 |
| Other gastroprotective agents | 956 (46.3) | 1146 (45) | 0.03 | 701 (46.6) | 695 (46.2) | 0.01 |
| Proton pump inhibitor | 1356 (65.7) | 1402 (55.1) | 0.22 | 915 (60.8) | 894 (59.4) | 0.03 |
| H_2_ receptor antagonist | 1601 (77.6) | 1911 (75.1) | 0.06 | 1143 (75.9) | 1133 (75.3) | 0.02 |
| Vitamin K therapy | 46 (2.2) | 153 (6) | 0.19 | 46 (3.1) | 40 (2.7) | 0.02 |
| Prasugrel | 0 (0) | 3 (0.1) | 0.05 | 0 (0) | 0 (0) | N/A |
| Alpha blockers | 385 (18.7) | 438 (17.2) | 0.04 | 265 (17.6) | 259 (17.2) | 0.01 |
| Antihypertensives | 901 (43.7) | 1070 (42) | 0.03 | 627 (41.7) | 630 (41.9) | 0.00 |
| Number of days on antihypertensives during baseline | 89.3 (86.1) | 84.7 (86.1) | 0.05 | 87.9 (86.5) | 85.9 (86.2) | 0.02 |
| **Procedure or tests, n (%)** |  |  |  |  |  |  |
| Previous cardiac procedure | 15 (0.7) | 37 (1.5) | 0.07 | 11 (0.7) | 9 (0.6) | 0.02 |
| Cardiovascular stress test | 15 (0.7) | 21 (0.8) | 0.01 | 10 (0.7) | 9 (0.6) | 0.01 |
| Echocardiogram | 734 (35.6) | 609 (23.9) | 0.26 | 469 (31.2) | 460 (30.6) | 0.01 |
| Colonoscopy | 177 (8.6) | 194 (7.6) | 0.04 | 111 (7.4) | 115 (7.6) | 0.01 |
| Fecal occult blood (FOB) test | 251 (12.2) | 164 (6.4) | 0.20 | 165 (11) | 156 (10.4) | 0.02 |
| Mammogram | 41 (2) | 46 (1.8) | 0.01 | 27 (1.8) | 31 (2.1) | 0.02 |
| Prostate-specific antigen test or Prostate exam for DRE | 157 (7.6) | 106 (4.2) | 0.15 | 119 (7.9) | 116 (7.7) | 0.01 |

**Abbreviations:** aPTT, Activated partial thromboplastin time; INR, International normalized ratio; DRE, Digital rectal examination.

Table S12. Baseline characteristics of EINSTEIN-DVT emulation cohort

| **Variables** | **Unmatched cohort** | |  | **PS Matched cohort** | |  |
| --- | --- | --- | --- | --- | --- | --- |
|  | **Rivaroxaban**  **(N=6,368)** | **Warfarin**  **(N=2,700)** | **aSD** | **Rivaroxaban**  **(N=2,135)** | **Warfarin**  **(N=2,135)** | **aSD** |
| **Median days of follow-up, (IQR)** |  |  |  | 49 (23-162) | 109 (30-249) |  |
| **Age, mean (SD), y** | 66.1 (15.2) | 62.7 (17.2) | 0.21 | 63.3 (16.7) | 62.9 (17.1) | 0.02 |
| **Sex, n (%)** | 2421 (38) | 1254 (46.4) | 0.17 | 941 (44.1) | 960 (45) | 0.02 |
| **Health status relevant with the use of anticoagulant, mean (SD)** |  |  |  |  |  |  |
| CHA_2_DS_2_ VASc score | 2.2 (1.5) | 2 (1.5) | 0.16 | 2 (1.6) | 2 (1.5) | 0.03 |
| HAS-BLED Score | 2.3 (1.1) | 2 (1) | 0.31 | 2 (1.1) | 2 (1) | 0.01 |
| **Number of laboratory tests, mean (SD)** |  |  |  |  |  |  |
| Number of brain natriuretic peptide tests | 0.1 (0.3) | 0.1 (0.3) | 0.01 | 0.1 (0.3) | 0.1 (0.3) | 0.00 |
| Number of cardiac biomarkers tests | 0.7 (1) | 0.8 (1) | 0.14 | 0.8 (1.1) | 0.8 (0.9) | 0.02 |
| Number of ambulatory blood pressure monitoring tests | 0.1 (0.4) | 0.1 (0.4) | 0.05 | 0.1 (0.4) | 0.1 (0.4) | 0.01 |
| Number of D-dimer tests | 0.3 (0.6) | 0.1 (0.5) | 0.28 | 0.2 (0.5) | 0.1 (0.5) | 0.01 |
| Number of C-reactive protein, high-sensitivity CRP tests | 0.8 (1.7) | 0.3 (1) | 0.39 | 0.3 (1) | 0.3 (1) | 0.01 |
| Number of prothrombin time or aPTT tests | 0.6 (1.2) | 0.2 (0.8) | 0.39 | 0.3 (0.8) | 0.3 (0.8) | 0.01 |
| Number of Bleeding time tests | 0 (0.2) | 0 (0.1) | 0.17 | 0 (0.1) | 0 (0.1) | 0.01 |
| Number of INR tests | 0 (0) | 0 (0.1) | 0.07 | 0 (0) | 0 (0) | N/A |
| Number of lipid tests | 0.3 (0.8) | 0.2 (0.6) | 0.25 | 0.2 (0.6) | 0.2 (0.6) | 0.01 |
| **Comorbidity, n (%)** |  |  |  |  |  |  |
| Deep vein thrombosis only | 6368 (100) | 2700 (100) | N/A | 2135 (100) | 2135 (100) | N/A |
| Pulmonary embolism with or without deep vein thrombosis | 0 (0) | 0 (0) | N/A | 0 (0) | 0 (0) | N/A |
| Previous venous thromboembolism | 961 (15.1) | 724 (26.8) | 0.29 | 528 (24.7) | 517 (24.2) | 0.01 |
| Active cancer | 808 (12.7) | 272 (10.1) | 0.08 | 248 (11.6) | 234 (11) | 0.02 |
| Hemorrhagic stroke or other cerebrovascular disease or cerebrovascular procedure | 361 (5.7) | 235 (8.7) | 0.12 | 161 (7.5) | 166 (7.8) | 0.01 |
| Major trauma potentially causing prolonged immobilization | 89 (1.4) | 25 (0.9) | 0.04 | 23 (1.1) | 21 (1) | 0.01 |
| Chronic kidney disease Stage 1-2 | 5 (0.1) | 1 (0) | 0.02 | 1 (0) | 1 (0) | 0.00 |
| Chronic kidney disease Stage 3-6 | 27 (0.4) | 15 (0.6) | 0.02 | 13 (0.6) | 12 (0.6) | 0.01 |
| Acute kidney injury | 59 (0.9) | 48 (1.8) | 0.07 | 34 (1.6) | 37 (1.7) | 0.01 |
| Bladder stones or kidney stones | 79 (1.2) | 39 (1.4) | 0.02 | 36 (1.7) | 31 (1.5) | 0.02 |
| Alcohol abuse/dependence or drug abuse/dependence | 28 (0.4) | 17 (0.6) | 0.03 | 11 (0.5) | 12 (0.6) | 0.01 |
| Atherosclerosis or cardiac conduction disorders or Other CVD | 633 (9.9) | 343 (12.7) | 0.09 | 259 (12.1) | 242 (11.3) | 0.03 |
| Diabetes with complication | 553 (8.7) | 234 (8.7) | 0.00 | 173 (8.1) | 169 (7.9) | 0.01 |
| Systemic embolism | 14 (0.2) | 11 (0.4) | 0.03 | 5 (0.2) | 7 (0.3) | 0.02 |
| Coagulation defects | 79 (1.2) | 33 (1.2) | 0.00 | 26 (1.2) | 28 (1.3) | 0.01 |
| Diabetes | 956 (15) | 399 (14.8) | 0.01 | 313 (14.7) | 311 (14.6) | 0.00 |
| Intracranial or retroperitoneal hemorrhage | 0 (0) | 0 (0) | N/A | 0 (0) | 0 (0) | N/A |
| Peptic ulcer disease | 187 (2.9) | 99 (3.7) | 0.04 | 67 (3.1) | 76 (3.6) | 0.02 |
| Upper gastrointestinal bleed | 0 (0) | 0 (0) | N/A | 0 (0) | 0 (0) | N/A |
| Lower/ unspecified gastrointestinal bleed | 0 (0) | 0 (0) | N/A | 0 (0) | 0 (0) | N/A |
| Urogenital bleed | 0 (0) | 0 (0) | N/A | 0 (0) | 0 (0) | N/A |
| Other bleeds | 0 (0) | 0 (0) | N/A | 0 (0) | 0 (0) | N/A |
| **Concomitant medication, n (%)** |  |  |  |  |  |  |
| Meglitinides | 15 (0.2) | 16 (0.6) | 0.06 | 9 (0.4) | 7 (0.3) | 0.02 |
| Alpha glucosidase inhibitors | 48 (0.8) | 33 (1.2) | 0.05 | 19 (0.9) | 23 (1.1) | 0.02 |
| Thiazide | 1186 (18.6) | 468 (17.3) | 0.03 | 373 (17.5) | 358 (16.8) | 0.02 |
| Beta blockers | 1126 (17.7) | 521 (19.3) | 0.04 | 423 (19.8) | 389 (18.2) | 0.04 |
| Calcium channel blockers | 2475 (38.9) | 925 (34.3) | 0.10 | 748 (35) | 738 (34.6) | 0.01 |
| All antidiabetic medications except Insulin | 1000 (15.7) | 426 (15.8) | 0.00 | 319 (14.9) | 325 (15.2) | 0.01 |
| Insulin | 693 (10.9) | 296 (11) | 0.00 | 220 (10.3) | 232 (10.9) | 0.02 |
| Low Intensity Statins | 75 (1.2) | 53 (2) | 0.06 | 39 (1.8) | 32 (1.5) | 0.03 |
| High Intensity Statins | 96 (1.5) | 73 (2.7) | 0.08 | 38 (1.8) | 48 (2.2) | 0.03 |
| Central nervous system stimulants | 20 (0.3) | 8 (0.3) | 0.00 | 4 (0.2) | 3 (0.1) | 0.01 |
| Estrogens or progestins or androgens | 187 (2.9) | 78 (2.9) | 0.00 | 63 (3) | 71 (3.3) | 0.02 |
| Angiogenesis inhibitors | 39 (0.6) | 7 (0.3) | 0.05 | 7 (0.3) | 5 (0.2) | 0.02 |
| Oral Immunosuppressants | 182 (2.9) | 115 (4.3) | 0.08 | 72 (3.4) | 72 (3.4) | 0.00 |
| Other direct thrombin inhibitors | 1 (0) | 0 (0) | 0.02 | 0 (0) | 0 (0) | N/A |
| Clopidogrel | 419 (6.6) | 287 (10.6) | 0.15 | 197 (9.2) | 192 (9) | 0.01 |
| Antibiotics | 5442 (85.5) | 2222 (82.3) | 0.09 | 1789 (83.8) | 1784 (83.6) | 0.01 |
| Aspirin | 1383 (21.7) | 779 (28.9) | 0.17 | 593 (27.8) | 561 (26.3) | 0.03 |
| Other antiplatelet agents | 1801 (28.3) | 775 (28.7) | 0.01 | 597 (28) | 588 (27.5) | 0.01 |
| P-glycoprotein inhibitors | 850 (13.3) | 401 (14.9) | 0.04 | 309 (14.5) | 298 (14) | 0.02 |
| Other gastroprotective agents | 2902 (45.6) | 1154 (42.7) | 0.06 | 940 (44) | 901 (42.2) | 0.04 |
| Proton pump inhibitor | 3030 (47.6) | 1056 (39.1) | 0.17 | 832 (39) | 840 (39.3) | 0.01 |
| H_2_ receptor antagonist | 5216 (81.9) | 2042 (75.6) | 0.15 | 1660 (77.8) | 1657 (77.6) | 0.00 |
| Vitamin K therapy | 283 (4.4) | 137 (5.1) | 0.03 | 90 (4.2) | 105 (4.9) | 0.03 |
| Prasugrel | 2 (0) | 1 (0) | 0.00 | 0 (0) | 0 (0) | N/A |
| Alpha blockers | 914 (14.4) | 383 (14.2) | 0.01 | 305 (14.3) | 304 (14.2) | 0.00 |
| Antihypertensives | 1882 (29.6) | 845 (31.3) | 0.04 | 650 (30.4) | 650 (30.4) | 0.00 |
| Number of days on antihypertensives during baseline | 83.2 (86.2) | 71.9 (84.7) | 0.13 | 74.1 (84.6) | 71.7 (84.7) | 0.03 |
| **Procedure or tests, n (%)** |  |  |  |  |  |  |
| Previous cardiac procedure | 18 (0.3) | 15 (0.6) | 0.04 | 8 (0.4) | 8 (0.4) | 0.00 |
| Cardiovascular stress test | 24 (0.4) | 14 (0.5) | 0.02 | 5 (0.2) | 9 (0.4) | 0.03 |
| Echocardiogram | 491 (7.7) | 182 (6.7) | 0.04 | 156 (7.3) | 150 (7) | 0.01 |
| Colonoscopy | 409 (6.4) | 218 (8.1) | 0.06 | 163 (7.6) | 164 (7.7) | 0.00 |
| Fecal occult blood (FOB) test | 213 (3.3) | 38 (1.4) | 0.13 | 31 (1.5) | 35 (1.6) | 0.02 |
| Mammogram | 114 (1.8) | 56 (2.1) | 0.02 | 49 (2.3) | 44 (2.1) | 0.02 |
| Prostate-specific antigen test or Prostate exam for DRE | 176 (2.8) | 40 (1.5) | 0.09 | 33 (1.5) | 35 (1.6) | 0.01 |

**Abbreviations:** aPTT, Activated partial thromboplastin time; INR, International normalized ratio; DRE, Digital rectal examination.

Table S13. Baseline characteristics of EINSTEIN-PE emulation cohort

| **Variables** | **Unmatched cohort** | |  | **PS Matched cohort** | |  |
| --- | --- | --- | --- | --- | --- | --- |
|  | **Rivaroxaban**  **(N=9,376)** | **Warfarin**  **(N=4,101)** | **aSD** | **Rivaroxaban**  **(N=2,801)** | **Warfarin**  **(N=2,801)** | **aSD** |
| **Median days of follow-up, (IQR)** |  |  |  | 105 (30-191) | 117 (28-278) |  |
| **Age, mean (SD), y** | 68.7 (15.9) | 68.9 (15.1) | 0.01 | 69 (15.5) | 69 (15.2) | 0.01 |
| **Sex, n (%)** | 3797 (40.5) | 1707 (40.7) | 0.01 | 1134 (40.5) | 1167 (41.7) | 0.02 |
| **Health status relevant with the use of anticoagulant, mean (SD)** |  |  |  |  |  |  |
| CHA_2_DS_2_ VASc score | 2.4 (1.5) | 2.5 (1.5) | 0.04 | 2.5 (1.5) | 2.5 (1.5) | 0.01 |
| HAS-BLED Score | 2.2 (1) | 2.3 (1) | 0.06 | 2.3 (1) | 2.3 (1) | 0.00 |
| **Number of laboratory tests, mean (SD)** |  |  |  |  |  |  |
| Number of brain natriuretic peptide tests | 0.2 (0.5) | 0.2 (0.5) | 0.02 | 0.2 (0.5) | 0.2 (0.5) | 0.02 |
| Number of cardiac biomarkers tests | 1.3 (1.1) | 1.1 (1) | 0.15 | 1.2 (1.1) | 1.2 (1) | 0.02 |
| Number of ambulatory blood pressure monitoring tests | 0.4 (0.6) | 0.3 (0.6) | 0.01 | 0.3 (0.6) | 0.3 (0.6) | 0.02 |
| Number of D-dimer tests | 0.5 (0.8) | 0.1 (0.5) | 0.58 | 0.2 (0.6) | 0.2 (0.6) | 0.03 |
| Number of C-reactive protein, high-sensitivity CRP tests | 1 (1.8) | 0.3 (1) | 0.53 | 0.4 (1.2) | 0.4 (1.2) | 0.02 |
| Number of prothrombin time or aPTT tests | 0.8 (1.3) | 0.2 (0.8) | 0.54 | 0.3 (0.9) | 0.3 (0.9) | 0.01 |
| Number of Bleeding time tests | 0 (0.2) | 0 (0.1) | 0.14 | 0 (0.1) | 0 (0.1) | 0.01 |
| Number of INR tests | 0 (0) | 0 (0) | 0.05 | 0 (0) | 0 (0) | N/A |
| Number of lipid tests | 0.5 (1) | 0.1 (0.6) | 0.48 | 0.2 (0.6) | 0.2 (0.7) | 0.00 |
| **Comorbidity, n (%)** |  |  |  |  |  |  |
| Deep vein thrombosis only | 0 (0) | 0 (0) | N/A | 0 (0) | 0 (0) | N/A |
| Pulmonary embolism with or without deep vein thrombosis | 9376 (100) | 4191 (100) | N/A | 2801 (100) | 2801 (100) | N/A |
| Previous venous thromboembolism | 1239 (13.2) | 672 (16) | 0.08 | 415 (14.8) | 432 (15.4) | 0.02 |
| Active cancer | 1503 (16) | 501 (12) | 0.12 | 419 (15) | 431 (15.4) | 0.01 |
| Hemorrhagic stroke or other cerebrovascular disease or cerebrovascular procedure | 505 (5.4) | 229 (5.5) | 0.00 | 164 (5.9) | 151 (5.4) | 0.02 |
| Major trauma potentially causing prolonged immobilization | 118 (1.3) | 50 (1.2) | 0.01 | 30 (1.1) | 29 (1) | 0.00 |
| Chronic kidney disease Stage 1-2 | 10 (0.1) | 6 (0.1) | 0.01 | 2 (0.1) | 1 (0) | 0.02 |
| Chronic kidney disease Stage 3-6 | 37 (0.4) | 33 (0.8) | 0.05 | 18 (0.6) | 14 (0.5) | 0.02 |
| Acute kidney injury | 104 (1.1) | 49 (1.2) | 0.01 | 34 (1.2) | 34 (1.2) | 0.00 |
| Bladder stones or kidney stones | 126 (1.3) | 47 (1.1) | 0.02 | 28 (1) | 31 (1.1) | 0.01 |
| Alcohol abuse/dependence or drug abuse/dependence | 104 (1.1) | 46 (1.1) | 0.00 | 31 (1.1) | 30 (1.1) | 0.00 |
| Atherosclerosis or cardiac conduction disorders or Other CVD | 1429 (15.2) | 794 (18.9) | 0.10 | 503 (18) | 495 (17.7) | 0.01 |
| Diabetes with complication | 772 (8.2) | 366 (8.7) | 0.02 | 223 (8) | 229 (8.2) | 0.01 |
| Systemic embolism | 8 (0.1) | 2 (0) | 0.02 | 0 (0) | 0 (0) | N/A |
| Coagulation defects | 86 (0.9) | 39 (0.9) | 0.00 | 26 (0.9) | 23 (0.8) | 0.01 |
| Diabetes | 1372 (14.6) | 659 (15.7) | 0.03 | 418 (14.9) | 432 (15.4) | 0.01 |
| Intracranial or retroperitoneal hemorrhage | 0 (0) | 0 (0) | N/A | 0 (0) | 0 (0) | N/A |
| Peptic ulcer disease | 337 (3.6) | 156 (3.7) | 0.01 | 107 (3.8) | 111 (4) | 0.01 |
| Upper gastrointestinal bleed | 0 (0) | 0 (0) | N/A | 0 (0) | 0 (0) | N/A |
| Lower/ unspecified gastrointestinal bleed | 0 (0) | 0 (0) | N/A | 0 (0) | 0 (0) | N/A |
| Urogenital bleed | 0 (0) | 0 (0) | N/A | 0 (0) | 0 (0) | N/A |
| Other bleeds | 0 (0) | 0 (0) | N/A | 0 (0) | 0 (0) | N/A |
| **Concomitant medication, n (%)** |  |  |  |  |  |  |
| Meglitinides | 19 (0.2) | 32 (0.8) | 0.08 | 18 (0.6) | 15 (0.5) | 0.01 |
| Alpha glucosidase inhibitors | 77 (0.8) | 46 (1.1) | 0.03 | 27 (1) | 31 (1.1) | 0.01 |
| Thiazide | 1677 (17.9) | 906 (21.6) | 0.09 | 587 (21) | 576 (20.6) | 0.01 |
| Beta blockers | 2409 (25.7) | 1245 (29.7) | 0.09 | 837 (29.9) | 812 (29) | 0.02 |
| Calcium channel blockers | 4123 (44) | 1896 (45.2) | 0.03 | 1291 (46.1) | 1287 (45.9) | 0.00 |
| All antidiabetic medications except Insulin | 1597 (17) | 697 (16.6) | 0.01 | 447 (16) | 457 (16.3) | 0.01 |
| Insulin | 1261 (13.4) | 642 (15.3) | 0.05 | 394 (14.1) | 405 (14.5) | 0.01 |
| Low Intensity Statins | 91 (1) | 48 (1.1) | 0.02 | 35 (1.2) | 31 (1.1) | 0.01 |
| High Intensity Statins | 371 (4) | 200 (4.8) | 0.04 | 108 (3.9) | 114 (4.1) | 0.01 |
| Central nervous system stimulants | 27 (0.3) | 17 (0.4) | 0.02 | 14 (0.5) | 7 (0.2) | 0.04 |
| Estrogens or progestins or androgens | 229 (2.4) | 120 (2.9) | 0.03 | 77 (2.7) | 73 (2.6) | 0.01 |
| Angiogenesis inhibitors | 52 (0.6) | 14 (0.3) | 0.03 | 17 (0.6) | 12 (0.4) | 0.03 |
| Oral Immunosuppressants | 219 (2.3) | 106 (2.5) | 0.01 | 69 (2.5) | 68 (2.4) | 0.00 |
| Other direct thrombin inhibitors | 5 (0.1) | 0 (0) | 0.03 | 0 (0) | 0 (0) | N/A |
| Clopidogrel | 1123 (12) | 610 (14.6) | 0.08 | 380 (13.6) | 411 (14.7) | 0.03 |
| Antibiotics | 8116 (86.6) | 3589 (85.6) | 0.03 | 2412 (86.1) | 2421 (86.4) | 0.01 |
| Aspirin | 2294 (24.5) | 1428 (34.1) | 0.21 | 898 (32.1) | 884 (31.6) | 0.01 |
| Other antiplatelet agents | 2984 (31.8) | 1394 (33.3) | 0.03 | 913 (32.6) | 943 (33.7) | 0.02 |
| P-glycoprotein inhibitors | 2030 (21.7) | 958 (22.9) | 0.03 | 635 (22.7) | 643 (23) | 0.01 |
| Other gastroprotective agents | 4389 (46.8) | 1979 (47.2) | 0.01 | 1359 (48.5) | 1336 (47.7) | 0.02 |
| Proton pump inhibitor | 5532 (59) | 2138 (51) | 0.16 | 1521 (54.3) | 1521 (54.3) | 0.00 |
| H_2_ receptor antagonist | 7256 (77.4) | 3320 (79.2) | 0.04 | 2191 (78.2) | 2179 (77.8) | 0.01 |
| Vitamin K therapy | 269 (2.9) | 260 (6.2) | 0.16 | 122 (4.4) | 122 (4.4) | 0.00 |
| Prasugrel | 6 (0.1) | 8 (0.2) | 0.04 | 4 (0.1) | 4 (0.1) | 0.00 |
| Alpha blockers | 1649 (17.6) | 741 (17.7) | 0.00 | 475 (17) | 481 (17.2) | 0.01 |
| Antihypertensives | 4075 (43.5) | 2001 (47.7) | 0.09 | 1331 (47.5) | 1304 (46.6) | 0.02 |
| Number of days on antihypertensives during baseline | 90.2 (86) | 93.2 (86.2) | 0.04 | 92.9 (85.6) | 93 (86) | 0.00 |
| **Procedure or tests, n (%)** |  |  |  |  |  |  |
| Previous cardiac procedure | 59 (0.6) | 44 (1) | 0.05 | 24 (0.9) | 30 (1.1) | 0.02 |
| Cardiovascular stress test | 105 (1.1) | 75 (1.8) | 0.06 | 28 (1) | 28 (1) | 0.00 |
| Echocardiogram | 2473 (26.4) | 562 (13.4) | 0.33 | 520 (18.6) | 503 (18) | 0.02 |
| Colonoscopy | 820 (8.7) | 347 (8.3) | 0.02 | 241 (8.6) | 249 (8.9) | 0.01 |
| Fecal occult blood (FOB) test | 647 (6.9) | 87 (2.1) | 0.24 | 75 (2.7) | 81 (2.9) | 0.01 |
| Mammogram | 156 (1.7) | 78 (1.9) | 0.02 | 52 (1.9) | 48 (1.7) | 0.01 |
| Prostate-specific antigen test or Prostate exam for DRE | 432 (4.6) | 49 (1.2) | 0.21 | 46 (1.6) | 49 (1.7) | 0.01 |

**Abbreviations:** aPTT, Activated partial thromboplastin time; INR, International normalized ratio; DRE, Digital rectal examination.

Table S14. Sensitivity analyses of intention-to-treat approach on the effectiveness and safety of RWE emulation study

|  | **Total Patients** | **Number of Events (%)** | **Incidence Rate*** | **Effect estimates**  **(95% CI)** |
| --- | --- | --- | --- | --- |
| **Sensitivity analyses 1:** Intention-to-treat approach^†^ | | | | |
| **AMPLIFY** | | | | |
| Recurrent VTE or related death | | | | |
| Warfarin | 1,753 | 90 (5.13) | 11.8 | 1.00 (Ref) |
| Apixaban | 1,753 | 44 (2.51) | 5.80 | 0.73 (0.65 to 0.83) |
| Major bleeding | | | | |
| Warfarin | 1,753 | 19 (1.08) | 2.44 | 1.00 (Ref) |
| Apixaban | 1,753 | 21 (1.20) | 2.76 | 1.05 (0.76 to 1.46) |
| **RE-COVER II** | | | | |
| Recurrent VTE or related death | | | | |
| Warfarin | 1,226 | 70 (5.71) | 12.8 | 1.00 (Ref) |
| Dabigatran | 1,226 | 46 (3.75) | 8.26 | 0.65 (0.45 to 0.94) |
| Major bleeding | | | | |
| Warfarin | 1,226 | 9 (0.73) | 1.60 | 1.00 (Ref) |
| Dabigatran | 1,226 | 7 (0.57) | 1.23 | 0.77 (0.29 to 2.06) |
| **Hokusai-VTE** | | | | |
| Recurrent VTE or related death | | | | |
| Warfarin | 1,505 | 95 (6.31) | 8.12 | 1.00 (Ref) |
| Edoxaban | 1,505 | 56 (3.72) | 4.79 | 0.59 (0.42 to 0.82) |
| Major bleeding | | | | |
| Warfarin | 1,505 | 20 (1.33) | 1.67 | 1.00 (Ref) |
| Edoxaban | 1,505 | 16 (1.06) | 1.35 | 0.81 (0.42 to 1.56) |
| **EINSTEIN-DVT** | | | | |
| Recurrent VTE | | | | |
| Warfarin | 2,135 | 66 (3.09) | 3.46 | 1.00 (Ref) |
| Rivaroxaban | 2,135 | 49 (2.30) | 2.55 | 0.74 (0.51 to 1.07) |
| Major bleeding | | | | |
| Warfarin | 2,135 | 30 (1.41) | 1.54 | 1.00 (Ref) |
| Rivaroxaban | 2,135 | 25 (1.17) | 1.29 | 0.84 (0.49 to 1.42) |
| **EINSTEIN-PE** | | | | |
| Recurrent VTE | | | | |
| Warfarin | 2,801 | 122 (4.36) | 5.09 | 1.00 (Ref) |
| Rivaroxaban | 2,801 | 85 (3.03) | 3.59 | 0.70 (0.53 to 0.93) |
| Major bleeding | | | | |
| Warfarin | 2,801 | 47 (1.68) | 1.92 | 1.00 (Ref) |
| Rivaroxaban | 2,801 | 36 (1.29) | 1.50 | 0.78 (0.50 to 1.20) |

**Abbreviations:** CI, Confidence interval; VTE, Venous thromboembolism

^*^Crude incidence rate per 100 person-years

^†^The same specified follow-up period of the trial was applied: 6 months for AMPLIFY and RE-COVER II, and 12 months for Hokusai-VTE, EINSTEIN-DVT, and EINSTEIN-PE.

Table S15. Sensitivity analyses of asymmetrical trimming and adjusting for PS deciles on the effectiveness and safety of RWE emulation study

|  | **Total Patients** | **Number of Events (%)** | **Incidence Rate*** | **Effect estimates**  **(95% CI)** |
| --- | --- | --- | --- | --- |
| **Sensitivity analyses 2:** Asymmetrical trimming of 2.5th percentile in the pre-matched cohort and adjusting for deciles of the PS | | | | |
| **AMPLIFY** | | | | |
| Recurrent VTE or related death | | | | |
| Warfarin | 3,451 | 149 (3.88) | 5.56 | 1.00 (Ref) |
| Apixaban | 3,839 | 106 (2.76) | 4.96 | 0.67 (0.52 to 0.85) |
| Major bleeding | | | | |
| Warfarin | 3,451 | 42 (1.22) | 1.55 | 1.00 (Ref) |
| Apixaban | 3,839 | 35 (0.91) | 1.62 | 0.86 (0.55 to 1.36) |
| **RE-COVER II** | | | | |
| Recurrent VTE or related death | | | | |
| Warfarin | 3,128 | 110 (7.64) | 7.55 | 1.00 (Ref) |
| Dabigatran | 1,439 | 38 (2.64) | 8.47 | 0.72 (0.49 to 1.04) |
| Major bleeding | | | | |
| Warfarin | 3,128 | 20 (0.64) | 1.35 | 1.00 (Ref) |
| Dabigatran | 1,439 | 7 (0.49) | 1.52 | 1.00 (0.41 to 2.49) |
| **Hokusai-VTE** | | | | |
| Recurrent VTE or related death | | | | |
| Warfarin | 2,482 | 83 (4.13) | 7.24 | 1.00 (Ref) |
| Edoxaban | 2,012 | 38 (1.89) | 5.73 | 0.56 (0.38 to 0.82) |
| Major bleeding | | | | |
| Warfarin | 2,482 | 18 (0.73) | 1.54 | 1.00 (Ref) |
| Edoxaban | 2,012 | 11 (0.55) | 1.65 | 0.92 (0.42 to 2.01) |
| **EINSTEIN-DVT** | | | | |
| Recurrent VTE | | | | |
| Warfarin | 2,633 | 56 (0.90) | 3.57 | 1.00 (Ref) |
| Rivaroxaban | 6,209 | 62 (1.00) | 3.92 | 0.66 (0.45 to 0.96) |
| Major bleeding | | | | |
| Warfarin | 2,633 | 25 (0.95) | 1.56 | 1.00 (Ref) |
| Rivaroxaban | 6,209 | 25 (0.40) | 1.56 | 1.09 (0.58 to 2.07) |
| **EINSTEIN-PE** | | | | |
| Recurrent VTE | | | | |
| Warfarin | 4,087 | 148 (1.62) | 5.50 | 1.00 (Ref) |
| Rivaroxaban | 9,142 | 129 (1.41) | 4.13 | 0.54 (0.42 to 0.69) |
| Major bleeding | | | | |
| Warfarin | 4,087 | 62 (1.52) | 2.23 | 1.00 (Ref) |
| Rivaroxaban | 9,142 | 80 (0.88) | 2.54 | 0.86 (0.60 to 1.23) |

**Abbreviations:** CI, Confidence interval; VTE, Venous thromboembolism

^*^Crude incidence rate per 100 person-years
